# Role of lysosomes in physiological activities, diseases, and therapy

**DOI:** 10.1186/s13045-021-01087-1

**Published:** 2021-05-14

**Authors:** Ziqi Zhang, Pengfei Yue, Tianqi Lu, Yang Wang, Yuquan Wei, Xiawei Wei

**Affiliations:** Laboratory of Aging Research and Cancer Drug Target, State Key Laboratory of Biotherapy, National Clinical Research Center for Geriatrics, West China Hospital, Sichuan University, No. 17, Block 3, Southern Renmin Road, Chengdu, 610041 Sichuan People’s Republic of China

**Keywords:** Lysosome, Atherosclerosis, Neurodegenerative disease, Pancreatitis, Autoimmune disorder, Lysosomal storage disorder, Tumor microenvironment, Tumor-associated macrophage

## Abstract

Long known as digestive organelles, lysosomes have now emerged as multifaceted centers responsible for degradation, nutrient sensing, and immunity. Growing evidence also implicates role of lysosome-related mechanisms in pathologic process. In this review, we discuss physiological function of lysosomes and, more importantly, how the homeostasis of lysosomes is disrupted in several diseases, including atherosclerosis, neurodegenerative diseases, autoimmune disorders, pancreatitis, lysosomal storage disorders, and malignant tumors. In atherosclerosis and Gaucher disease, dysfunction of lysosomes changes cytokine secretion from macrophages, partially through inflammasome activation. In neurodegenerative diseases, defect autophagy facilitates accumulation of toxic protein and dysfunctional organelles leading to neuron death. Lysosomal dysfunction has been demonstrated in pathology of pancreatitis. Abnormal autophagy activation or inhibition has been revealed in autoimmune disorders. In tumor microenvironment, malignant phenotypes, including tumorigenesis, growth regulation, invasion, drug resistance, and radiotherapy resistance, of tumor cells and behaviors of tumor-associated macrophages, fibroblasts, dendritic cells, and T cells are also mediated by lysosomes. Based on these findings, a series of therapeutic methods targeting lysosomal proteins and processes have been developed from bench to bedside. In a word, present researches corroborate lysosomes to be pivotal organelles for understanding pathology of atherosclerosis, neurodegenerative diseases, autoimmune disorders, pancreatitis, and lysosomal storage disorders, and malignant tumors and developing novel therapeutic strategies.

## Background

### Overview of lysosomes

Lysosomes are composed of acid lumen and a layer of lysosomal membrane formed by phospholipid bilayer. The acid lumen contains a host of hydrolytic enzymes, including nucleases, proteases, phosphatases, lipases, sulfatases, and others. These enzymes are synthesized in endoplasmic reticulum, and modified in Golgi, where the proteins are added mannose-6-phosphate residue, a tag targeting lysosomes [1]. On lysosomal membranes, the overarching protein maintaining acid luminal pH is vacuolar H+-ATPase (v-ATPase) that hydrolyses adenosine triphosphate (ATP) to transport protons into lysosomes [2]. Other key lysosomal membrane proteins include several ion channels, through which ions move inward/outward [3]; lysosome-associated membrane proteins (LAMPs), including LAMP1-5, among which LAMP1 and LAMP2 are well documented to be involved in phagocytosis, chaperone-mediated autophagy (CMA), macroautophagy, lipid transport, and aging [4]; soluble N-ethylmaleimide-sensitive factor activating protein receptors (SNAREs) and RAB GTPases that are involved in trafficking and fusion [5]; toll-like receptors (TLRs) that sense pathogen-associated molecular patterns (PAMPs) and initiate inflammatory response [6]; and mammalian target of rapamycin (mTOR) that co-ordinates several homeostatic signaling pathways [7]. Notably, a series of terms have been proposed to classify lysosomes, such as terminal lysosomes, endolysosomes, protolysosomes, phagolysosomes, and autolysosomes indicating heterogeneity of lysosomes. In a word, the complex lysosomal luminal and membrane proteins indicate lysosomes to be pleiotropic organelles.

Lysosomes are formed through fusion of endosomes and vesicles budded from *trans*-Golgi network. The main regulator of lysosomal biogenesis is microphthalmia/transcription factor E (MiT/TFE) protein family including transcription factor EB (TFEB), transcription factor EC (TFEC), transcription factor binding to IGHM enhancer 3 (TFE3), and melanocyte inducing transcription factor (MITF) [8], among which TFEB is the best studied. TFEB can be phosphorylated on several sites by a number of kinases and phosphatases, such as mechanistic target of rapamycin complex 1 (mTORC1) on Ser211 [9], Ser142 [10], and Ser122 [11], glycogen synthase kinase 3β (GSK3β) on Ser134 and Ser138 [12], extracellular signal-regulated kinase (ERK) on Ser142 [13], mitogen-activated protein kinase kinase kinase kinase 3 (MAP4K3) on Ser3 [14], and protein kinase B (PKB) on Ser467 [15] repressing nuclear translocation of TFEB. Dephosphorylation of TFEB by calcineurin [16] and protein phosphatase 2 (PP2A) [17] lets TFEB enter nuclear to regulate transcription. For example, if nutrient is in abundance, TFEB is phosphorylated by mTORC1, which facilitates 14-3-3-dependent retention of TFEB in cytoplasm and prevents transcription function of TFEB [9]. On the contrary, in the context of starvation or mTOR inhibitor treatment, inactivated mTOR leads to accumulation of dephosphorylated TFEB resulting in nuclear translocation of TFEB [10]. Alternatively, in some cases, lysosomes release Ca^2+^ through transient receptor potential mucolipin 1 (TRPML1) to activate calcineurin that dephosphorylates TFEB [16]. In nucleus, TFEB transcribes coordinated lysosomal expression and regulation (CLEAR) network genes that code proteins highly related to structure and function of lysosomes [18] including receptors and enzymes participating in lysosomal sorting. Both soluble and membrane lysosomal proteins should be recognized by sorting receptors. The best-known sorting receptor is the mannose-6-phosphate receptors (M6PRs) that recognize M6P-tagged proteins in the Golgi complex [19]. Newly synthesized lysosomal proteins are successively modified by oligosaccharyltransferase [20], GlcNAc-1-phosphotransferase, and uncovering enzyme [21] to be tagged with M6P residues. In Golgi complex with pH 6.7, tagged proteins bind with M6PRs, while in acidic endosomes with pH 6, the proteins are released [19]. Additionally, a subgroup of M6PRs, cation-independent MPR, is able to localize at plasma membrane, which underpins retrieving of escaped extracellular lysosomal proteins [19]. Besides M6PRs, two sorting receptors are also found, sortilin and lysosomal integral membrane protein 2 (LIMP-2). Sortilin is involved in the sorting of prosaposin [22], acid sphingomyelinase [23], cathepsin D, and cathepsin H [24]. LIMP-2 mediates the transport of β-glucocerebrosidase [25], whose dysfunction is associated with Gaucher disease (GD). For lysosomal membrane proteins, tyrosine-based or dileucine-based signals are essential for their lysosomal targeting through adaptor proteins or Golgi-localising, Gamma-adaptin ear domain homology, ARF-binding proteins (GGAs) [26, 27]. Early endosomes budding from post-Golgi complex engage multiple rounds of membrane fusion and fission, resulting in formation of late endosomes and lysosomes [28, 29]. In a word, the lysosomal biogenesis pathway is accurately controlled to keep the homeostasis of cell.

### Function of lysosomes

Recent discoveries have changed the former consideration of lysosomes as organelles that degrade and recycle cellular waste. It is now clear that lysosomes are key organelles in degradation, innate and adaptive immunity, and nutrient sensing [28]. Tempting researches have highlighted many signal pathways interacting with lysosomal activities manifesting the central role of lysosomes in many physiological processes [29]. In addition, progressions of several diseases are also regulated by lysosomes [30].

#### Autophagy

Lysosomal degradation is classified as endocytosis and autophagy that is composed of macroautophagy, microautophagy, and CMA. Cytoplasmic materials, like mitochondria, are recycled through macroautophagy triggered by Unc-51-like kinase (ULK) complex and phosphatidylinositol 3-kinase (PI3K) complex, during which double-membrane phagophores are formed and then mature into autophagosomes [31]. To undergo degradation, autophagosomes then fuse with lysosomes with help of microtubule-associated protein 1A/B light chain 3B (LC3B)-I/II [32]. Microautophagy is described as a concise process that lysosome directly engulfs cytosolic material via invagination of lysosomal membrane under the mediation of vacuolar protein sorting 4 (VPS4) and ALG-2 interacting protein X (ALIX) [33]. In CMA, cytosolic soluble proteins are recognized through KFERQ-like motif by complex containing members of heat shock proteins (HSPs), HSPA8/HSC70. Then the proteins are translocated into the lysosomal lumen with help of lysosome-associated membrane protein type 2A (LAMP2A) for degradation [34]. At present, the mechanisms of autophagy have been intensively studied, thus demonstrating autophagy dysfunction to be a crucial step in the pathology of many diseases and a target for interventions.

#### Innate and adaptive immunity

Immunity is directly impacted by lysosomal activities in cells like DCs and macrophages. For innate immunity, pathogens like bacteria are internalized through phagocytosis and targeted to lysosomes for degradation. In cases of bacteria that are able to escape from phagosomes into cytosol, autophagy presents an additional mechanism that captures and delivers bacteria for lysosomal degradation [35, 36]. Additionally, several TLRs on lysosomal membrane are able to recognize various microbial and host-derived ligands and elicit pro-inflammatory signals [6]. For adaptive immunity, antigenic peptides are generated for presentation by major histocompatibility complex class II (MHC-II) molecules to CD4^+^ T cells. During this process, tubulation of phagosomes and lysosomes elicited by TLR4 signaling is important for antigen presentation [37, 38]. Interestingly, autophagy displays the opposite role in MHC-II and MHC-I antigen presentation. Transfer of cytosolic proteins into lysosomes and MHC-II presentation is promoted by autophagy [39], while inhibition of autophagy downregulates MHC-II presentation [40]. On the contrary, autophagy augments major histocompatibility complex class I (MHC-I) internalization resulting in dampened MHC-I presentation [41], which is mainly based on transporter associated with antigen processing complex. A better understanding of the innate and adaptive immunity pathway would be quite important for developing immunotherapy for cancer.

#### Amino acid sensing

Another function of lysosomes is amino acid sensing dependent on the localization of mTORC1 complex to lysosomal membrane. Activation of Ragulator and Rag GTPases mediated by amino acid is the key for lysosomal membrane localization of mTORC1 complex [42]. Solute carrier family 38 member 9 (SLC38A9), an amino acid transporter and sensor, putatively senses arginine [43], glutamine, and leucine [44] to activate mTORC1 through Ragulator and Rag GTPases. Additional sensing components that are involved in mTORC1 activation are Sestrin2 for leucine [45] and cellular arginine sensor for mTORC1 (CASTOR) for arginine [46], respectively. When activated, mTOR functions as the master regulator of various cellular behaviors such as growth, autophagy, and lysosomal biogenesis. It’s still unknown whether other nutrient sensing mechanisms on lysosomal membranes exist.

In brief, lysosomes are the hub of several crucial cellular activities and signals in physiological conditions. In addition, extra lysosome-related mechanisms have been reported in diseases including atherosclerosis, neurodegenerative diseases, pancreatitis, autoimmune disorders, lysosomal storage disorders, and cancer. There are some interesting similarities among these mechanisms. Detailed understanding of these mechanisms inspires the development of therapies targeting lysosomes.

## Role of lysosomes in non-malignant diseases

### Lysosomes in atherosclerosis

The development of atherosclerosis may result in coronary syndromes [47], ischaemic strokes [48], intermittent claudication [49], and aneurysms [50], owing to plaque growth and thrombus formation. Low-density lipoprotein cholesterol (LDL-C) and oxidized LDL (oxLDL) [51] engulfed by macrophage-derived foam cells accumulation in the intima of arteries is the key during atherosclerosis, which causes chronic inflammation [52]. Afterward, these cells may engage programmed cell death resulting in a necrotic core covered by a fibrous cap, whose rupturing exposes tissue factor and provokes thrombosis [53]. As the main source of foam cells, macrophages have been rigorously studied, in which lysosomes are demonstrated to be involved in almost the whole process of atherosclerosis.

During the initiation of atherosclerosis, membrane scavenger receptors such as CD36 [54, 55], scavenger receptor A (SR-A) [55], and lipoprotein receptor-1 (LOX-1) [56] are responsible for the internalization of lipids, especially oxLDL, into lysosomes. The internalized LDL is hydrolyzed by enzymes like lysosomal acid lipase to produce free cholesterol that is exported to endoplasmic reticulum for re-esterification and storage. Substantial decrease in lysosomal acid lipase activity leads to development of premature atherosclerosis in human [57]. The lipid processing course can be in trouble when macrophages are exposed to oxLDL [54]. Treatment with oxLDL or cholesterol crystal caused lysosomal dysfunction featured with elevated lysosomal pH, increased lysosomal membrane permeability, diminished degradation capacity, and morphological changes [58]. Together, dysfunction of lysosomes owing to oxLDL or decreased lipase may lead to pathological changes that cause atherosclerosis through regulating autophagy, inflammasomes, apoptosis, and lysosomal biogenesis.

Autophagy has shown its protective role in atherosclerosis development through promoting lipid droplet hydrolysis and eventual efflux of free cholesterol from foam cells [59]. Inhibition of autophagy by autophagy-related 5 *(Atg5)* deletion accelerated atherosclerotic plaque development [60]. Unfortunately, atherosclerotic plaque development did impair autophagy conversely in macrophages [61, 62] accounting for cholesterol crystal accumulation and dysfunctional mitochondria, which are responsible for inflammasome hyperactivation, M1-like polarization, and apoptosis [63]. The cholesterol crystal accumulation activated NOD-like receptor family, pyrin domain containing 3 (NLRP3) inflammasome that releases interleukin-1 beta (IL-1β) [62] through lysosomal leakage containing cathepsins [54]. Impaired autophagy also caused dysfunctional mitochondria that triggered apoptosis through reactive oxygen species (ROS) and cytochrome C [63]. In addition, it has been proved in other disease models that cathepsins from lysosomes are also the inducer of apoptosis in cytoplasm [64, 65]. Apoptosis of macrophages, or foam cells, is the key step in the formation of lipid-rich necrotic core [53]. In a word, both activated inflammasome and apoptosis in macrophages are crucial for atherosclerotic plaque development.

The aforementioned deteriorations initiate several signal changes, which are largely based on activated mTOR indirectly by cholesterol via a lysosomal transmembrane protein SLC38A9 [66]. Activated mTOR may start several detrimental mechanisms. First, mTOR activation inhibits autophagy, whose suppression causes damages described in the foregoing paragraph. Second, cholesterol trafficking from lysosomes to endoplasmic reticulum is obstructed by activated mTOR [67]. Third, activated mTOR inhibits nuclear translocation of TFEB [9, 10] that reverses lysosomal dysfunction [58]. Therapeutic benefits of mTOR disruption by Cre/loxP recombination [68] or small molecular inhibitors [69] are in accordance with these mechanisms. What’s more, although detailed mechanisms are still fuzzy, the association between mTOR and macrophage polarization are proposed [70, 71]. Inhibiting mTOR elevated M2-like polarization that stabilizes plaques and hampered M1-like polarization that promotes plaque rupture [70, 72–74]. It’s noteworthy that a more complex grouping of macrophages in plaques has been put forward based on single-cell RNA sequencing [75]. A better understanding of mTOR-related pathways is tempting for mediation of atherosclerotic plaque development.

**Fig. 1:**
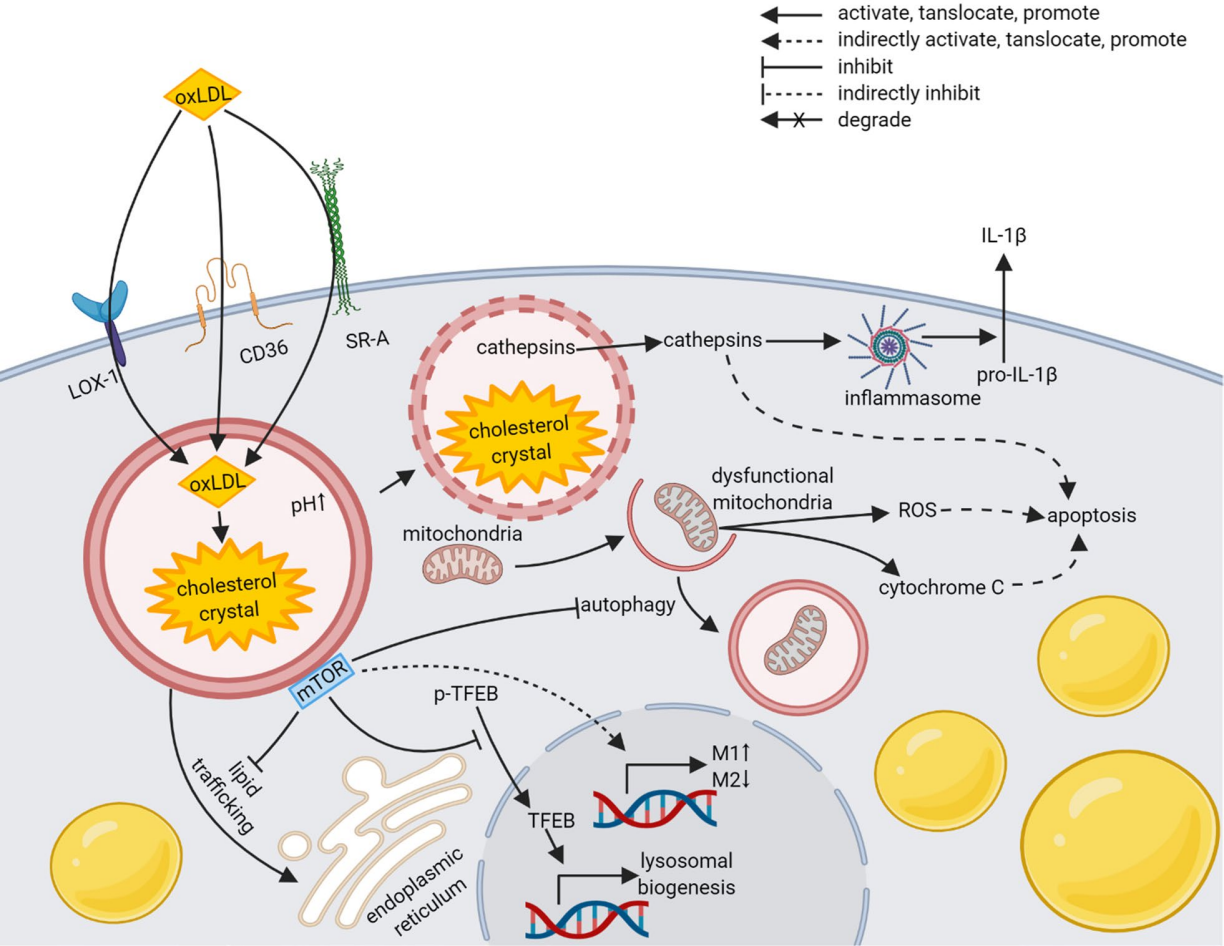
Role of lysosomes in development of atherosclerosis. Created with BioRender.com

In summary, lysosomes in macrophages are involved in initiation, development, and progression of atherosclerotic plaque, which have been exhibited in Fig. 1 and Table 1. They are the crucial nodes linking lipid degradation, autophagy, apoptosis, inflammasome, lysosomal biogenesis, and macrophage polarization. Researches on lysosomes and mTOR signal are promising for prevention and therapy of atherosclerosis, several of which have inspired lysosome-targeting clinical trials listed in Table 4.

**Table 1:** Diseases related with lysosomal dysfunction

Diseases	Lysosomal dysfunction	Outcomes	Reference
*Atherosclerosis*	Lysosomal acid lipase deficient	Substantial decrease in lysosomal acid lipase activity leads to premature atherosclerosis in human	[57]
	OxLDL or cholesterol crystal accumulation	OxLDL or cholesterol crystal causes lysosomal membrane permeability, autophagy deficient, mitochondrial dysfunction, inflammasome activation, and apoptosis	[54, 58, 61–63]
*Neurodegeneration diseases*			
Alzheimer's disease	*Presenilin 1* mutation	Defective Presenilin 1-dependent lysosomal acidification is one of the main causes of early-onset familial AD	[93–95, 340, 341]
	*Becn1* ablation	Heterozygous deletion of beclin 1 (Becn1) results in autophagy disruption, Aβ deposition, and neurodegeneration	[97]
	*Cathepsin D* mutation	The T-allele of cathepsin D rs17571 increases risk of AD	[92]
	*Cathepsin B* ablation	Ablation of cathepsin B increases the abundance of Aβ42 and potentiates plaque deposition	[91]
Parkinson’s disease	*Snca* mutation	A53T point mutation in the *Snca* gene causes familial PD	[107, 108]
	*Atp13a2* ablation	*Atp13a2* depletion leads to lysosomal membrane instability, impaired acidification, blocked clearance of autophagosomes, α-syn accumulation, and cell death	[109, 110]
	*Gba1* mutation	Mutations in the *Gba1* gene are important risk factors for PD	[113]
Huntington disease	*Htt* mutation	Mutated HTT protein has abnormally long polyglutamine (polyQ) repeats near the N-terminus, which promotes formation of toxic oligomers and neuronal inclusion bodies	[119, 120]
	*Wdfy3* ablation	Depletion of *Wdfy3* accelerates the accumulation of polyQ aggregates	[127]
	*Sqstm1* knockdown	*Sqstm1* knockdown increases mHTT-induced cell death	[128]
*Pancreatitis*	Impaired autophagy flux	Increased autophagosome formation and decreased autophagosome clearance are observed	[140, 342]
	Imbalanced cathepsin B and cathepsin L	Imbalance between cathepsin B and cathepain L contributes to accumulation of activated intracellular trypsin	[140]
*Autoimmune disorders*			
Systemic lupus erythematosus	Enhanced autophagy in T cells	Enhanced autophagy causes imbalanced T cell subsets	[146–148]
	Defect LC3-associated phagocytosis	Defect LC3-associated phagocytosis leads to blunted clearance of dying cells and elevated inflammation	[149]
	Defect lysosomal acidification	Macrophages in lupus shows elevated lysosomal pH	[343]
Crohn’s disease	Defect autophagy	Human or mice deficient in ATG16L1 are more susceptible to Crohn’s disease	[150, 151]
	Defect lysosomal acidification	Elevated luminal pH links lysosomal dysfunction with Crohn’s disease risk	[344]
Rheumatoid arthritis	Impaired autophagy	Reduced autophagy links altered metabolism and T cell exhaustion	[152]
Multiple sclerosis	Enhanced autophagy	Enhanced ATG5 expression in T cells is correlated with more sever disability	[153]
*Lysosomal storage disorder*			
Niemann-Pick type C (NPC) disease	*Npc1* or *Npc2* mutation	Defective NPC1 or NPC2 causes lysosomal accumulation of cholesterol and glycosphingolipids leading to hepatic, pulmonary, and neuropsychiatric disorder	[345]
Fabry disease	*Galactosidase α* mutation	*Galactosidase α* mutation causes globotriaosylceramide accumulation in lysosomes leading to vascular diseases	[346]
Tay-Sachs disease	*β-hexosaminidase α* mutation	Deficient β-hexosaminidase α causes GM2 ganglioside accumulation in lysosomes of nerve cells leading to neuro disorder	[347]
Mucopolysaccharidoses diseases	Mutation in mucopolysaccharide catabolic enzymes	Lysosomal accumulation of mucopolysaccharides leads to disorders in bone, cartilage, connective tissues, and nervous	[348]
Pompe disease	*α glucosidase* mutation	Mutated α glucosidase causes glycogen accumulation in lysosomes leading to cardiac and respiratory failure	[349]
Gaucher disease	*glucosylceramidase β* mutation	Glucosylceramide accumulates in macrophage lysosomes leading to disorder in visceral organs and nervous system	[162]

### Lysosomes in neurodegenerative diseases

Aggregation of abnormal proteins and organelles is common in neurodegenerative diseases. The autophagy-lysosome pathway (ALP) is essential for the survival of non-dividing neurons by contributing to the removal of abnormal large protein aggregates and organelles [76, 77]. Defective clearance by ALP and/or increased abnormal protein aggregation is common in the pathogenesis of several neurodegenerative diseases, including Alzheimer's disease (AD), Parkinson’s Disease (PD), and Huntington disease (HD) [78]. It is difficult to determine whether the defect of ALP is the cause or result of neurodegeneration. Indeed, aggregation of toxic proteins, impaired organelles, oxidative stress, and insufficient ATP can interact with defective ALP leading to a worse situation and eventual cell death [79, 80]. Several genetic defects and lysosome-targeting clinical trials have been summarized in Tables 1 and 4, respectively. Here we summarily review the role of lysosomal dysfunction in progressive neurodegenerative disorders.

#### Alzheimer's disease

The pathology of AD is defined by the deposition of amyloid-beta (Aβ) peptides in extracellular amyloid plaques and the accumulation of neurofibrillary tangles in cells formed by phosphorylated microtubule-associated protein tau [81]. The dysfunction of ALP has been proposed to play an important role in AD [82].

Amyloid precursor protein (APP) can be cleaved through the non-amyloidogenic pathway or the amyloidogenic pathway. The non-amyloidogenic pathway excludes the formation of Aβ [83, 84], while the amyloidogenic pathway produces Aβ40 and Aβ42, which tend to misfold to form aggregates [85]. If lysosomal function is impaired or Aβ production increases, Aβ will accumulate in neurons leading to cell death and the pathological manifestations of AD [86]. Tau, a microtubule-associated phosphoprotein, is normally localized in the neuronal axon, where it promotes microtubule assembly and stabilizes microtubules. In AD, ALP dysfunction promotes tau aggregation and causes hyperphosphorylated tau that accumulate and form neurofibrillary tangles in neurons [87, 88] promoting neurodegenerative development in AD patients [89]. Additionally, the hyperphosphorylation of tau may in turn cause autophagy dysfunction owing to the instability of neuronal microtubules, which affects the position and function of lysosomes [90].

Genetic variations causing dysfunction in ALP pathway are important in the pathogenesis of AD. In mice, genetic inactivation of cathepsin B that cleaves Aβ42 increased Aβ42 and potentiated plaque deposition, while cathepsin B over-expression reduced the deposition of pre-existing amyloid protein [91]. Consistent with this, in a population-based cohort study, the T allele of cathepsin D rs17571 was associated with an increased risk of AD [92]. *Presenilin 1 (PS1)* mutation is one of the main causes of early-onset familial AD [93, 94]. The *PS1* mutant impedes v-ATPase V0a1 subunit resulting in impaired proton pump function, increased lysosomal pH, affected lysosomal proteolysis, and destroyed autophagy [95]. The expression of Beclin 1, an initiator of autophagy pathway, in AD brain is reduced [96]. The deficient Beclin 1 in vitro and *vivo* reduced neuronal autophagy resulting in the deposition of Aβ, while its overexpression mitigated the accumulation of Aβ [97]. Other attempts to restore ALP [98], such as TFEB transfection [99], have yield promising therapeutic effects.

In summary, these genetic variations deter the clearance of abnormal proteins in neurons by lysosomes, indicating restoring LAP in neurons to be a potential therapeutic strategy.

#### Parkinson’s disease

The accumulation of misfolded proteins plays a central role in the pathogenesis in PD and impairs lysosomal function [100]. Accumulation of aggregated proteins in Lewy bodies, which are mainly composed of alpha­synuclein (α-syn), is the major pathogenic event in PD [101]. Both CMA pathway and macroautophagy pathway are important for mediating α-syn degradation in neurons [102], while lysosomal dysfunction leads to α-syn aggregation [103–105]. Inhibition of either system leads to increased levels of α-syn [106].

The A53T point mutation in the *Snca* gene that encodes α-syn causes familial PD via accelerated oligomerization or fibrillization of the protein [107, 108]. Other alterations in lysosome-related genes impair the degradation of misfolded proteins. ATPase cation transporting 13A2 (ATP13A2) depletion leads to α-syn accumulation through lysosomal dysfunction [109, 110]. Mutations in lysosomal hydrolases are also critical risk factors for the disease [111, 112]. For instance, mutated glucocerebrosidase, commonly found in Gaucher disease, is important risk factor for PD [113]. Activating autophagy by Latrepirdine [114] or TFEB overexpression [13, 115] showed promising therapeutic effects in preclinical researches.

In a word, genetic alterations promote PD through both accelerating α-syn accumulation and decelerating α-syn clearance, and enhancing autophagy is a possible strategy.

#### Huntington disease

HD is a rare autosomal­dominant neurodegenerative disease caused by detrimental aggregation­prone HTT mutants (mHTT) with an aberrant expansion of cysteine, adenine, and guanine (CAG) trinucleotide repeats within exon 1 of the HTT gene [116–118]. The mHTT has abnormally long polyglutamine (polyQ) repeats near the N-terminus, which promotes the formation of toxic oligomers and aggregates to form nuclear and cytoplasmic neuronal inclusion bodies [119, 120]. Because wild-type huntingtin serves as a scaffold for the recruitment of several autophagy proteins [121], accumulation of mHTT induces lysosomal and autophagy dysfunctions.

Interestingly, HD neurons show an increased number of autophagosomes and maintain appropriate (or even higher) levels of autophagy flux [122, 123]. Although the initiation of autophagy and the formation of autophagic vesicles increased through inactivating mTOR [124], the aggregated mHTT and damaged organelles will be seldom transferred to autophagosomes resulting in their accumulation in the cytoplasm and toxicity [123]. Macroautophagy plays a key role in the clearance of mutant HTT aggregates [125, 126], during which adaptor protein, autophagy-linked FYVE protein (ALFY), is necessary for clearing mHTT aggregation [127]. Depletion of ALFY accelerated the accumulation of aggregates, while increasing ALFY expression reduced protein aggregates and mitigated polyQ toxicity [127]. In addition, dysfunction of p62, an autophagy receptor protein, significantly increased neuron death induced by mHTT [128]. Notably, when macroautophagy is impaired by mHTT, compensatory CMA activity may be up-regulated suggesting the existence of crosstalk among autophagy pathways [129]. However, the compensation mechanism may decrease with age, leading to neuronal loss and the pathological manifestations of HD [130]. Rapamycin and rapamycin analog CCI-779 (temsirolimus) can effectively attenuate the accumulation of huntingtin protein and improve the motor performance in the mice [124].

Thus, developing therapies that upregulate autophagy is likely to relieve mHTT aggregation in patients.

### Lysosomes in pancreatitis

Present researches shed light on the involvement of lysosomes in pathophysiology of pancreatitis [131]. Normally, pancreatic acinar cells secrete digestive enzymes, many of which are inactive zymogens packed in zymogen granules until they reach the intestines [132]. A canonical hypothesis for pancreatitis is intra-acinar trypsinogen activation [131]. Excessive secretagogue-receptor or bile salt receptor activation causes abnormal peak-plateau calcium signal, which disrupts zymogen granule exocytosis, blocks secretion, and initiates the formation of endocytic vacuoles [133, 134]. The vacuoles containing trypsin and trypsinogen fuse with lysosomes containing cathepsin B that transforms trypsinogen into trypsin [131, 135]. Alternatively, macrophages phagocytose extracellular zymogen-containing vesicles and generate active trypsin [136]. The unstable endocytic vacuoles would rupture and release trypsin and cathepsin B into cytoplasm, thus provoking apoptosis or necrosis [137]. Additionally, calcium or tumor necrosis factor-α (TNF-α) from recruited leukocytes are able to initiate necroptosis in acinar cells [138, 139].

However, debates about the intra-acinar trypsinogen activation exist, because cathepsins co-localize with digestive enzymes in physiological autophagy without manifestation of pancreatitis [140]. Lysosomal dysfunction in pancreatitis is indicated by the findings that the maturation of cathepsin B and cathepsin L are reduced and that autophagosome formation is increased while lysosomal degradation is decreased [140]. Since cathepsin B converts trypsinogen to trypsin [141] while cathepsin L digests both trypsinogen and trypsin [142], the imbalance between cathepsin B and cathepsin L, especially insufficient cathepsin L maturation, may contribute to trypsin accumulation and development of pancreatitis [140]. Besides abnormal cathepsins, disruption of autophagy-related genes, *ATG5* [143], *ATG7* [144], or *LAMP2* [145], induces spontaneous or chronic pancreatitis implying the importance of autophagy in pancreatitis pathology.

In summary, the detailed involvement of lysosomal dysfunction and cathepsin activities in pancreatitis is still controversial. Quantification or semi-quantification of autophagy activities and cathepsin activities in patients or animal models may help clarify the mechanisms. The lysosomal dysfunction in pancreatitis has been summarized in Table 1.

### Lysosomes in autoimmune disorders

Multi-facetted role of lysosomes in autoimmune disorders has been demonstrated by the finding that autophagy, cathepsin expression, and luminal pH are altered in different immune cells in patients or mouse models [30]. In lupus, activated autophagy supports survival, development, and differentiation in B cells and T cells [146, 147]. Imbalance between pro-inflammatory Th17 and immunosuppressive regulatory T cells (Tregs) is attributed to autophagy activation in these T cell subsets [148]. However, mice deficient in LC3-associated phagocytosis, a non-canonical autophagy, showed blunted clearance of dying cells and elevated inflammatory cytokines, immune complex deposition, and autoantibodies as well as more sever lupus symptom [149]. Autophagy deficiency is also indicated in Crohn’s disease [150, 151] and T cells from rheumatoid arthritis [152], while intensified autophagy in T cells is highlighted in multiple sclerosis [153]. The regulation of glucocorticoid receptors by lysosomal degradation is a contentious topic that lysosome inhibitors, chloroquine or bafilomycin A1, enhance therapeutic effects of glucocorticoid in rheumatoid arthritis [154], while autophagy activator, rapamycin, declines prednisone requirement in lupus patients [155]. The above findings imply various autophagy alterations in different cell subsets from different autoimmune disorders. Thus, interventions targeting autophagy pathway should be carefully designed and tested. The lysosomal dysfunction and lysosome-targeting clinical trials for autoimmune disorders have been summarized in Tables 1 and 4, respectively.

### Lysosomes in lysosomal storage disorders (LSDs)

LSDs are a group of heterogeneous inherited metabolic disorders, most of which are attributed to mutations of particular lysosomal hydrolases. The pathology of LSDs can be explained by the accumulation of the substrates of the hydrolases or the secondary products formation instigated by the substrates [30]. An exception is mucolipidosis type IV, where mutated *MCOLN1*, a non-selective cation channel gene, impairs the fusion of lysosomes with both autophagosomes and late endosomes [156]. Common detrimental autophagy alterations and following changes have been revealed in LSDs. Deregulated mitophagy leads to increased damaged mitochondria engendering reactive oxygen species, ATP production, and imbalanced Ca^2+^ [157]. Microautophagy is impaired in Pompe disease [158]. Defective CMA caused by mutated *LAMP2* has been claimed in Danon disease [159]. Detailed descriptions of the deficient proteins in LSDs have been listed in Table 1. The enzyme replacement therapies for LSDs are underpinned by the mechanisms that some cation-independent MPRs are localized at plasma membrane to retrieve escaped extracellular lysosomal proteins [19]. Thus, supplying the appropriate enzymes is able to reverse the pathology of LADs. Alternatively, gene therapies are newly developed to restore deficient genes in patients. Clinical trials testing enzyme replacement therapies and gene therapies of LADs have been listed in Table 4. It will be an interesting topic to compare enzyme replacement therapies with gene therapies.

A special feature for GD is its relationship with increased risk for malignancies, particularly hematological malignancies [160, 161]. The biallelic *glucosylceramidase β* gene mutation causes glucocerebroside accumulation in lysosomes in macrophages, the Gaucher cells (GCs) [162] that conglomerate to form Gaucheromas in liver, spleen, bone marrow, and even nervous system [160, 161]. Patients with GD are more frequent to develop multiple myeloma and several hematological malignancies [161, 163]. Additionally, elevated frequencies of malignancies in kidney, liver, prostate, testis, brain, bone, colon, and melanoma are also related with GD [161, 164]. The predisposition of malignant tumor could be explained, at least partially, by an altered microenvironment related to phenotypes of GCs [160]. Impaired autophagy in GCs led to p65-nuclear factor-kappaB (NF-κB) activation following with IL-1β secretion by inflammasome pathway [165]. However, anti-inflammatory interleukin-13 (IL-13) was also increased [166]. Several works demonstrated that the phenotype of GCs resembled alternatively activated macrophages [167, 168]. In fact, M2-like GCs were surrounded by spleen cells with strong and frequent M1-like markers such as IL-1β and monocyte chemotactic protein-1 (MCP-1) [167]. We propose that the existence of both M1-like and M2-like macrophages in GD tissues resembles a sterile chronic inflammation environment facilitating malignancies development in various mechanisms [169–172]. In a word, abnormal lysosomal storage disorder in macrophages is closely related to macrophage activation that may create an inflammatory microenvironment suitable for tumorigenesis.

## Role of lysosomes in tumor microenvironment

Malignant tumor is an increasingly severe health problem making up the growing rate of death nowadays. In most cases, conventional therapies display only limited effects. The difficulties in therapy exist not only in complex tumor cells but also in complicated tumor microenvironment (TME) composed of macrophages, T cells, dendritic cells, fibroblasts, and other cells. It has been clear that the progression and regression of tumors are highly dependent on the dynamic interactions between various cells within TME [173]. In the microenvironment characterized by innutrition, acidity, hypoxia, and ischemia, tumor cells reprogram phenotypes and behaviors of stromal cells to promote tumor progression through inducing proliferation, drug resistance, invasion, metastasis, and immunosuppression [173]. These interactions are achieved through membrane proteins, cytokines, metabolites, exosomes, and others. Compelling results have substantiated lysosomes are exceedingly evolved in the phenotype and behavior changes. In the following paragraphs, lysosome-related mechanisms in several cell subgroups are introduced and these mechanisms are summarized in Table 3.

### Lysosomes in tumor cells

Massive knowledge about tumor cells has been collected over the past decades. Tumor cells are now considered as a group of cells that contain unstable genome with numerous mutations and are able to proliferate continuously, evade cell death, induce angiogenesis, invade, metastasize, avoid immune supervision, resistant to chemotherapy, and resistant to radiotherapy [174]. Paradigms such as epithelial-mesenchymal transition and cancer stem cells have been proposed to explain and link these malignant behaviors. Additionally, activities of lysosomes are implicated in the phenotypes of malignant cells. The overarching lysosomal activity, autophagy, has been shown to inhibit tumorigenesis, yet promote tumor progression. Autophagy prevents tumorigenesis by inhibiting the transformation of pre-malignant cells [175, 176]. Genotoxic ROS and dysfunctional mitochondria are eliminated by autophagy [177, 178]. Moreover, autophagy could process genomic instability, such as extruded cytoplasmic chromatin fragments [179], micronuclei [180], and endogenous retrotransposons [181]. However, elevation of autophagy has been shown in primary tumor cells and cell lines [182, 183]. Comparing the differences of autophagy between pre-malignant cells and cancer cells will be an interesting topic. Autophagy, as well as other lysosome-related mechanisms facilitate tumor progression by promoting malignant phenotypes mentioned above. Here we focus on the mechanisms through which lysosomes regulate proliferation, invasion, radiotherapy resistance, and chemotherapy resistance.

#### Regulation of tumor cell proliferation by lysosomes

Lysosomes regulate tumor cell proliferation through manipulating growth factor signals and providing nutrients. Growth factor signals are initiated at plasma membrane by receptor tyrosine kinases (RTK), which can be limited by endocytic degradation in lysosomes [184]. Epidermal growth factor receptor (EGFR), a member of RTK, can be internalized from plasma membrane to macropinocytic structures with help of adaptor molecule, growth factor receptor bound protein 2 (Grb2), when cells are stimulated with epidermal growth factor (EGF), the ligand of EGFR [185]. This feedback mechanism can be controlled by a tumor suppressor protein, receptor-associated late transducer (RALT) [186]. On the other hand, in order to sustain tumor growth, tumor cells need more nutrients than normal cells to provide energy and biosynthetic material [187]. Lysosomes degrade recycled intracellular materials and internalized extracellular proteins by autophagy and macropinocytosis, respectively, to supply amino acids [183, 187, 188]. The utilization of extracellular proteins for nutrient supplements is achieved when mTORC1 is inactivated [189]. As expected, inhibited autophagy and macropinocytosis impeded tumor growth [182, 187]. Moreover, Davidson et al. put the hypothesis forward in their review that autophagy may also be an adaptive strategy to salvage nucleotides by recycling endogenous and exogenous nucleic acids [174]. Collectively, lysosomes are the key regulator of both proliferation signal and proliferation materials. A good question is whether lysosomes are able to regulate other growth factor signals and provide other kinds of nutrients for tumor cells.

#### Regulation of tumor cell invasion by lysosomes

Invasion is one of the most crucial malignant behaviors, which jeopardizes adjacent normal tissues and is tightly related to metastasis. A key step of invasion is degradation of the extracellular matrix. Cathepsins, a family of peptidase in lysosome, have been extensively researched, because of their significant relationship with invasion, metastasis, and prognosis [190]. They are categorized into cysteine peptidases (cathepsins B, C, F, H, K, L, O, S, V, W, and X), serine peptidases (cathepsins A and G), and aspartic peptidases (cathepsins D and E) [65]. Expression alterations of cathepsins are ubiquitous in tumors, as summarized in Table 2. Both tumor cells and stromal cells are able to release cathepsins into TME; however, cathepsins from distinct cells may have different functions [191, 192]. In most cases, up-regulated cathepsins are associated with migration, invasion, and metastasis indicating tumor progression and poor prognosis. Thus, many of the cathepsins have been identified as prognostic markers or therapeutic targets. Cathepsin B, for example, is able to degrade laminin, collagen IV, and fibronectin, three major components of the basement membrane, at both acid and neutral pH [193]. To make matters worse, cystatins, endogenous cathepsin inhibitors, are down-regulated in some cancers [194]. Besides degradation of extracellular matrix, cathepsin B and X in several tumor cell lines are able to stimulate epithelial-mesenchymal transition (EMT), a process that has been wildly recognized to promote invasion [195]. Moreover, cathepsin B also promotes migration in glioma-initiating cells through mediating c-Jun amino terminal kinase (JNK) [196]. Consistent with these findings, administration of cathepsin inhibitors diminished tumor invasion [197]. Interestingly, although potentiated invasion and metastasis caused by elevated expression of cathepsins are wildly recognized, several reverse relationships between cathepsins and tumor progression have been reported (Table 3). Augmented cathepsin X was involved in early tumorigenesis of colon carcinoma, nonetheless, gradual loss of cathepsin X was detected during advanced local invasion and metastasis leading to poor prognostic outcome [198]. A similar pattern was found in cathepsin E whose expression was significantly higher in Barrett’s esophagus than normal tissue, but esophageal adenocarcinoma expressed a relatively lower cathepsin E level than Barrett’s esophagus [199]. Another favorable role of cathepsins in patients was seen in cathepsin V from thymic carcinoma [200] and cathepsin E from bladder cancer [201] and breast cancer [202]. The diverse effects of cathepsins on different tumors imply various unknown mechanisms. In fact, the roles of cathepsins in tumor are considerably beyond promoting invasion [197]. More details about the involved mechanisms would be helpful for understanding their different effects on tumor progression.

**Table 2:** Expression and significance of cathepsins in tumor cells and stromal cells

Type of cathepsin	Type of tumor	Type of samples and expression changes	Outcomes	Reference
*Cathepsin B*	Laryngeal cancer	Tissues (↑) Cell line (HEP-2)	Cathepsin B is positively correlated with migration, invasion, and proliferation	[350]
	Gastric cancer	Tissues (↑) Patient serum (↑)	Serum cathepsin B is positively associated with late stage and poor prognosis	[351]
	Breast cancer	Tissues (↑)	Cathepsin B is a prognostic marker for indication of recurrence	[352]
	Non-small cell lung cancer	Tissues (↑)	Activity of cathepsin B is significantly higher in tumor	[353]
	Melanoma	Patient serum (↑)	Serum cathepsin B indicates metastatic melanoma and shorter overall survival	[354]
	Colorectal cancer	Tissues (↑)	Elevated cathepsin B (from TAMs) is correlated with metastases	[355]
	Glioblastoma	Cell line (SNB19)	Cathepsin B and MMP-9 promote tumor invasion, growth, and angiogenesis	[356]
	Prostate cancer	Cell line (PC3 and DU145)	Cathepsin B and MMP-9 are positively correlated with cell survival, invasion, and angiogenesis	[357]
	Pancreatic islet cell carcinoma	Tissues from mouse model (RIP1-Tag2) (↑)	Cathepsin B promotes tumor formation, angiogenesis, invasion, and proliferation	[358]
	Meningioma	Tissues	Cathepsin B is expressed in endothelium and microvessels	[359]
*Cathepsin C*	Colorectal cancer	Cell lines (HCT-116, HT29, and KM12C)	Cathepsin C inhibition blocks autophagy and induced ER stress and apoptosis	[360]
	Renal cell carcinoma	Cell lines (786-O and A-498)	Timosaponin AIII suppresses cathepsin C expression, thus inhibiting cell migration and invasion	[361]
	Non-small cell lung cancer	Tissues (↑)	High cathepsin C expression is correlated with tumor recurrence	[362]
	Hepatocellular carcinoma	Tissues (↑) Cell lines (SK-HEP-1, SMMC-7721, hepg2, MHCC-97H, Hep3B, and PLC/PRF/5)	Cathepsin C promotes proliferation and metastasis	[363]
	Pancreatic islet cell carcinoma	Tissues from mouse model (RIP1-Tag2) (↑)	Cathepsin C is expressed in tumor tissues	[358]
	Squamous carcinoma	Tissues from mouse model (K14-HPV16) (↑)	Cathepsin C facilitates squamous carcinogenesis process	[364]
	Tongue cancer	Tissues	Cathepsin C is expressed in tongue cancer	[365]
*Cathepsin F*	Osteosarcoma	Cell lines (saos-2, lm5, and lm-7)	Cathepsin F is negatively correlated with metastasis	[366]
	Gastric cancer	Tissues (↓) Cell lines (SGC7901, BGC823, MGC803, HGC27, AGS, and MKN45) (↓)	Cathepsin F is negatively correlated with proliferation and cell survival	[367]
	Cervical cancer	Tissues (↑) Cell lines (hela, calo, INBL, siha, and caski) (↑)	Cathepsin F is involved in pathogenesis of cervical cancer	[368]
*Cathepsin H*	Hepatocellular carcinoma	Cell line (HepG2)	Cathepsin B is expressed in HepG2	[369]
	Pancreatic islet cell carcinoma	Mouse model (RIP1-Tag2)	Cathepsin H promotes tumor growth, angiogenesis, and decreases apoptosis	[370]
	Lung cancer	Tissues (↓) Patient serum (↑)	Serum cathepsin H is higher in patients	[371]
	Glioblastoma	Tissues (↑) Cell lines (u251, uwr1, and uwr2)	Cathepsin H promotes tumor cell invasion	[372]
	Prostate cancer	Cell line (PC-3)	Cathepsin H mediates migration and invasion	[373]
	Melanoma	Patient serum (↑)	Cathepsin H indicates shorter overall survival rates	[354]
	Basal cell carcinoma	Tissues (↑)	Cathepsin H (from peritumoral cells) promotes invasion of tumor	[374]
*Cathepsin K*	Glioblastoma	Expression data from database (↑)	Cathepsin K is not correlated with survival	[375]
	Breast cancer	Cell lines (MDA-MB-231 and SK-BR-3)	Cathepsin K promotes proliferation and metastasis	[376]
	Oral squamous cell carcinoma	Tissues (↑)	Cathepsin K (from stromal and tumor cells) indicates lymph node metastasis, perineural invasion, and poor survival	[377]
	Colorectal cancer	Tissues (↑) Cell line (MC38)	Cathepsin K (from tumor cells) promotes invasion, M2-like polarization of TAMs, and poor outcomes	[378]
	Prostate cancer	Tissues (↑) Cell lines (lncap, C4-2B, and PC3) (↑)	Cathepsin K promotes cell invasion	[379]
	Skull base chordoma	Tissues (↑)	Cathepsin K is correlated with reduced progression-free survival	[380]
	Ovarian cancer	Tissues (↑) Cell line (OV-2008)	Cathepsin K promotes metastasis and poor prognosis	[381]
	Melanoma	Tissues (↑)	Cathepsin K is an independent predictor of metastasis	[382]
	Gastric cancer	Tissues (↑)	Cathepsin K promotes tumor recurrence	[383]
*Cathepsin L*	Breast cancer	Cell line (MDA-MB-468 and MCF-7)	Cathepsin L is located in nucleus with help of Snail	[384]
	Glioma	Cell line (U87 and U251)	Cathepsin L promotes cell survival, migration, and invasion	[385]
	Cervical cancer	Tissues (↑) Cell lines (MS751, Caski, hela, C33A, and siha)	Cathepsin L facilitates migration and invasion of cancer cells	[386]
	Ovarian cancer	Tissues (↑) Cell lines (SKOV3 and SKOV3/TAX)	Cathepsin L promotes cell proliferation, migration, invasion, and paclitaxel resistance	[387]
	Gastric cancer	Tissues (↑)	High cathepsin L is correlated with metastases, poor differentiation, and diffuse histotype	[388]
	Colorectal cancer	Tissues (↑) Cell lines (SW480, SW620, SW1116, SW837, and SW948)	Cathepsin L is inversely associated with survival	[389]
	Non-small cell lung cancer	Cell lines (A549 and H1299)	Mutated *K-ras* promotes cathepsin L expression in irradiation treated cells resulting in enhanced invasion and migration	[390]
	Pancreatic cancer	Tissues (↑) patient serum (↑)	High serum cathepsin L is associated with poor prognosis	[391]
	Melanoma	Cell line (mv3)	Cathepsin L is detected in cell supernatants	[392]
*Cathepsin O*	Breast cancer	Tissues	A *Cathepsin O* mutation is correlated with shorter disease-free and overall survival	[393]
*Cathepsin S*	Prostate cancer	Tissues (↑) Mouse model (TRAMP)	Cathepsin S (from TAMs) is expressed in castration-resistant, poor differentiation, or high Gleason grade tumor	[394]
	Gastric cancer	Tissues (↑) Patient serum (↑) Cell lines (SGC7901, MKN45, AGS, MGC803) (↑)	Cathepsin S is correlated with higher TNM, later stage, and poorer overall survival	[395]
	Hepatocellular carcinoma	Cell line (MHCC97-H)	Cathepsin S inhibition induces apoptosis and chemosensitivity	[396]
	Triple-negative breast cancer	Cell lines (MDA-MB-231 and MCF-7)	Cathepsin S promotes cell growth and metastasis	[397]
	Papillary thyroid cancer	Expression data from database (↑)	Cathepsin S is a predictive marker for progression and prognosis	[398]
	Non-small cell lung cancer	Patient serum	Cathepsin S activity is detected in patient serum	[399]
	Colorectal cancer	Tissues (↑) Cell line (SL4)	Cathepsin S is associated with M2-like TAMs, higher histologic grade, and later clinical stage	[257]
*Cathepsin V/L2*	Colorectal cancer	Tissues (↑)	Cathepsin L2 is expressed in colorectal cancer	[400]
	Breast cancer	Tissues (↑)	High cathepsin V predicts poor prognosis	[401]
	Thymic carcinoma	Tissues	Cathepsin V inhibits tumor recurrences	[200]
	Endometrial cancer	Tissues (↑)	Cathepsin V expression is correlated with growth regulatory gene expression	[402]
*Cathepsin W*	–	–	No research about cathepsin W in tumor was found	–
*Cathepsin X*	Breast cancer	Cell line (MCF-7)	Cathepsin X participates in EMT	[195]
	Gastric cancer	Tissues Cell line (N87)	Cathepsin X inhibits G1 arrest and apoptosis	[403]
	Colorectal cancer	Tissues	Increased cathepsin X (from TAMs) is found during tumorigenesis; however, loss of cathepsin X is correlated with tumor progression	[198]
	Prostate cancer	Tissues (↑)	Cathepsin X is expressed in prostate cancer and prostatic intraepithelial neoplasia	[404]
	Glioblastoma	Expression data from database (↑)	High cathepsin X is correlated with poor survival	[375]
*Cathepsin A*	Prostate cancer	Tissues (↑) Cell lines (pc3 and du145)	Cathepsin A promotes proliferation, EMT, and tumorigenesis	[405]
	Melanoma	Tissues (↑) Cell lines (B78, MmB16, and B16F10)	High cathepsin A activity was detected in melanoma lesions	[406, 407]
	Lung cancer	Expression data from database (↑) Cell line (A549)	Cathepsin A promotes cell proliferation, migration, and invasion	[408]
	Colorectal cancer	Tissues (↑) Cell lines (HCT116 and lovo)	High cathepsin A is associated with lymph node and liver metastasis	[409]
	Breast cancer	Tissues (↑)	High cathepsin A indicates poor prognosis and shorter recurrence-free interval	[410]
*Cathepsin G*	Acute myeloid leukemia	Primary patient samples	Cathepsin G is a marker for poor survival	[411]
	Tongue squamous cell carcinoma	Tissues	Cathepsin G is expressed in peri-tumoral stroma	[412]
	Acute lymphoblastic leukemia	Primary patient samples Cell lines (SUP-B15, SB, RS4;11, NALM6, Raji, and T2)	Cathepsin G is a poor prognosticator	[413]
	Glioblastoma	Tissues	Cathepsin G is expressed in the microvasculature	[414]
	Meningioma	Tissues	Cathepsin G is expressed in the interstitium	[359]
*Cathepsin D*	Bladder cancer	Tissues	Cathepsin D is highly expressed in some tissue	[415]
	Osteosarcoma	Tissues (↑)	Cathepsin D is a biomarker for osteosarcomas and pulmonary metastases	[416]
	Breast cancer	Tissues (↑) Cell lines (MDA-MB-231)	High cathepsin D is correlated with shorter recurrence-free survival	[417]
	Endometrial cancer	Patient serum (↑)	Cathepsin D and IgG was detected in patients	[418]
	Hepatocellular carcinoma	Patient serum (↑)	Cathepsin D is detected in patient serum	[419]
	Prostate cancer	Cell line (PC-3)	Cathepsin D fosters cell proliferation and invasion	[420]
	Nasopharyngeal carcinoma	Tissues cell lines (6-10B, 5-8F, CNE2, and CNE1)	Down-regulated cathepsin D indicates poor histological differentiation, while up-regulated cathepsin D indicates metastasis and poor prognosis	[421]
	Pancreatic cancer	Cell line (MIApaca2)	Cathepsin D and pro-cathepsin D promote cancer cell dissemination	[422]
	Non-small cell lung cancer	Tissues	Cathepsin D together with caspase 3^−^ or p53^+^ are predictor for tumor node metastasis and lymph node metastasis, respectively	[423]
	Gastric cancer	Tissues	Cathepsin D participates in cancer metastasis	[383]
	Squamous cell carcinoma	Tissues (↑)	Cathepsin D is intensively expressed in poorly differentiated tissues	[424]
	Melanoma	Tissues (↑)	Cathepsin D is associated with tumor development	[425]
	Colorectal cancer	Patient serum (↑)	Cathepsin D is detected in patient serum	[426]
	Ovarian cancer	Tissues (↑)	Higher cathepsin D is expressed in more serous ovarian carcinoma. Cathepsin D in tumor epithelial cells may be beneficial prognostic factor	[427]
	Meningioma	Tissues	Cathepsin D is expressed in endothelium and microvessels	[359]
*Cathepsin E*	Pancreatic cancer	Tissues (↑)	Cathepsin E is detected in pancreatic cancer	[428]
	Esophageal cancer	Tissues (↑)	Barrett’s esophagus possesses higher cathepsin E than normal tissues. Esophageal cancer shows lower cathepsin E than Barrett’s esophagus but higher than normal tissues	[199]
	Bladder cancer	Tissues	High cathepsin E is correlated with better progression-free survival	[201]
	Gastric cancer	Tissues (↑)	Cathepsin E is a marker of signet-ring cell carcinoma and gastric differentiation	[429]
	Breast cancer	Patients serum	Cathepsin E is associated with favorable prognostic outcomes	[202]

↑ upregulation, ↓ downregulation

**Table 3:** Function of lysosomes in subset of cells in TME

Cell subset	Function of lysosomes	Outcomes	Reference
*Tumor cell*	Proliferation	EGFR can be internalized from plasma membrane and degrade in lysosomes	[184, 185]
		Autophagy and macropinocytosis provide extra nutrients	[183, 187, 188]
	Invasion	Cathepsins degrade extracellular component and promote EMT	[193, 195]
	Radioresistance	Radioresistance can be achieved through autophagy, cathepsins, and tumor stem cells	[205, 206, 212, 214, 215]
	Chemoresistance	Drugs are sequestrated in lysosomes by actively transportation or passive diffusion	[220]
		Autophagy promotes or inhibits cancer cell death in different situation	[232–237]
*TAMs*	M2-like polarization	Autophagy, LAP, and CMA regulate M2-like polarization at most cases	[257, 259, 260]
	M1-like polarization	TLRs on lysosomal membrane regulate M1-like polarization	[262, 264, 266]
		Elevated luminal pH is correlated with M1-like polarization	[275, 276]
	Invasion promotion	TAM-derived cathepsins promote tumor invasion	[280, 283, 284]
	Chemoresistance induction	TAM-derived cathepsins protect tumor cells from drug-induced apoptosis	[279]
	Infiltration promotion	TAM-derived cathepsins promote macrophage infiltration into TME	[290, 291]
*DCs*	Antigen presentation	LAMP3^+^ DCs are able to elicit CD8^+^ T cell immunity. Autophagy may promote or inhibit antigen presentation	[293–296]
*CAFs*	Chemoresistance regulation	Autophagy in CAFs promotes or inhibits chemoresistance in different tumor	[303, 304]
	Proliferation promotion	Autophagy in CAFs provides nutrients for tumor cells	[299, 300]
	Invasion promotion	Autophagy in CAFs promotes EMT in tumor cells	[305]
	Stemness promotion	CAFs with active autophagy release HMGB1 to enhance stemness in tumor cells	[306]
*T cells*	CD8^+^ T cell immunity	Autophagy in CD8^+^ T cells promotes or inhibits CD8^+^ T cell immunity in different tumor	[309, 310]
	CD4^+^ T cell immunity	Autophagy in CD4^+^ T cells inhibits anti-tumor effects by T_H_9 cell	[311, 312]
	Treg inhibition	Autophagy in Treg promotes survival and stability of Treg cells	[313]

#### Regulation of tumor cell radioresistance by lysosomes

Radiotherapy is now wildly used in the therapy of malignant tumors, whose therapeutic effect is based on direct DNA damage through radiation itself and indirect DNA damage through ROS and reactive nitrogen (RNS) species. Following DNA damage, cell death is induced in tumor cells. However, tumor cells sometimes develop radioresistance resulting in recrudesce and therapy failure. Several factors related to radioresistance have been revealed at present, including autophagy, cathepsins, and tumor stem cells. The autophagy response may vary depending on different dosages and kinds of radiation, as well as different tumor cells. More frequently, ionizing radiation induces autophagy [203] through mTOR inhibition [204] or autophagy-related 4B cysteine peptidase (ATG4B) [205] activation. Although detailed mechanisms are still ambiguous, the protective role of autophagy in tumor cells treated with radiation has been verified in various tumor cells, possibly depending on liver kinase B1 (LKB1) [206] and p53 [207]. In accordance with the reported protective role of autophagy, a series of attempts blocking autophagy resulted in increased radiosensitivity, including pharmacological inhibition, gene silencing, and gene knockout [203]. Therapy strategies combining radiotherapy and autophagy inhibition have been tested, which showed promising outcomes [208, 209]. Knockdown of TFEB, the downstream transcription factor of mTOR, diminished radioresistance in cancer cells, indicating the involvement of lysosomal biogenesis in autophagy-dependent radioresistance [210]. However, Kim et al. showed that radiation-activated mTOR and elicited de novo protein synthesis instead of autophagy [211] implying a context-dependent regulation of mTOR and autophagy. Besides autophagy, cathepsins also mediate radioresistance in tumor cells. Elevation of cathepsin B [212], S [213], and L [214] was observed in tumor cells after radiation treatment. Potential mechanism of cathepsin-induced radioresistance is related to cell cycle mediation and DNA repairment, because knockdown of cathepsin L led to G_2_/M phase cell cycle arrest [214] and decreased phosphorylation of DNA repair checkpoint protein, ataxia-telangiectasia-mutated kinase (ATM) and DNA-dependent protein kinase (DNA-PKcs) [215]. Nuclear translocation of NF-κB and following activation of cyclin D1 and ATM were highlighted as downstream signal of cathepsin L [216]. A link between cathepsins and tumor stem cells is another mechanism of radioresistance. Cathepsin B [212, 217] and L [218] have been shown to relate significantly with tumor stem cells, a subgroup of cells that are resistant to radiotherapy [219]. Cathepsin L knockdown dramatically reduced CD133, a stem cell marker [215]. In a word, knockdown or inhibition of cathepsins and autophagy are potential strategies to increase the radiosensitivity of tumor cells.

#### Regulation of tumor cell chemoresistance by lysosomes

An intriguing mechanism of lysosome-mediated chemoresistance in tumor cells has been well elaborated. Briefly, drugs can be sequestrated in lysosomes through passive diffusion or active transportation, which prevents drugs from reaching their intracellular targets [220]. For passive diffusion, lysosomes are distinguished by their luminal pH of about 4.5–5, hence lysosomes are able to sequester lipophilic, weakly basic drugs without the help of transporters [221, 222]. Once accumulate in lysosomes, these drugs can hardly pass the lysosomal membrane because of protonation [220]. Alternatively, chemotherapy drugs can also enter lysosomes through trafficking by P-glycoprotein (P-gp), an ATP-dependent efflux pump [221]. Normally, P-gp is embedded in the plasma membrane, but it can be incorporated into lysosomal membranes through intracellular trafficking [223]. Of note, acid lysosomal pH and P-gp may cooperate to achieve drug sequestration [224]. Several effectors are involved in the mediation of drug sequestration, including P-gp expression, luminal pH, and lysosomal biogenesis. Expression of P-gp can be up-regulated by hypoxia-inducible factor-1α (HIF-1α) elicited by stressors in the microenvironment [223]. In breast cancer cell line, drug-resistant MCF-7 cells appeared more acid luminal pH than normal MCF-7 cells [225]. As expected, increase in lysosomal pH and drug-sensitization were observed through knockdown of v-ATPase that maintains luminal acid pH [226]. Exposed to hydrophobic weak bases drugs, cancer cells showed an increased number of lysosomes per cell [227], indicating lysosomal biogenesis. The lysosomal biogenesis could be regulated by inhibition of mTOR and nucleus translocation of TFEB when weak base drugs accumulate in lysosomes [228]. Development of methods targeting drug sequestration may help improving the effect of chemotherapy.

It’s still in passionate debate whether autophagy should be inhibited or stimulated in tumor therapy. On the one hand, extensively activated autophagy can result in autophagic cell death [229]. On the other hand, massive evidence have highlighted autophagy as a process protecting cancer cells against chemotherapy [230, 231]. In pre-clinical researches, inhibition of autophagy flux enhanced anti-tumor effects of drugs against glioblastoma [232], breast cancer [233], gastric cancer, [234], and pancreatic cancer [235]. On the contrary, a positive correlation between autophagy and cell death has been found in squamous carcinoma [236] and lymphoblastic leukemia [237]. In clinical trials, both autophagy stimulator, rapamycin, and inhibitor, hydroxychloroquine are largely tested. Further results with quantification or semi-quantification, rather than stimulation/inhibition, of autophagy in cancer cells might be helpful in determining how interventions targeting autophagy should be administrated.

**Fig. 2:**
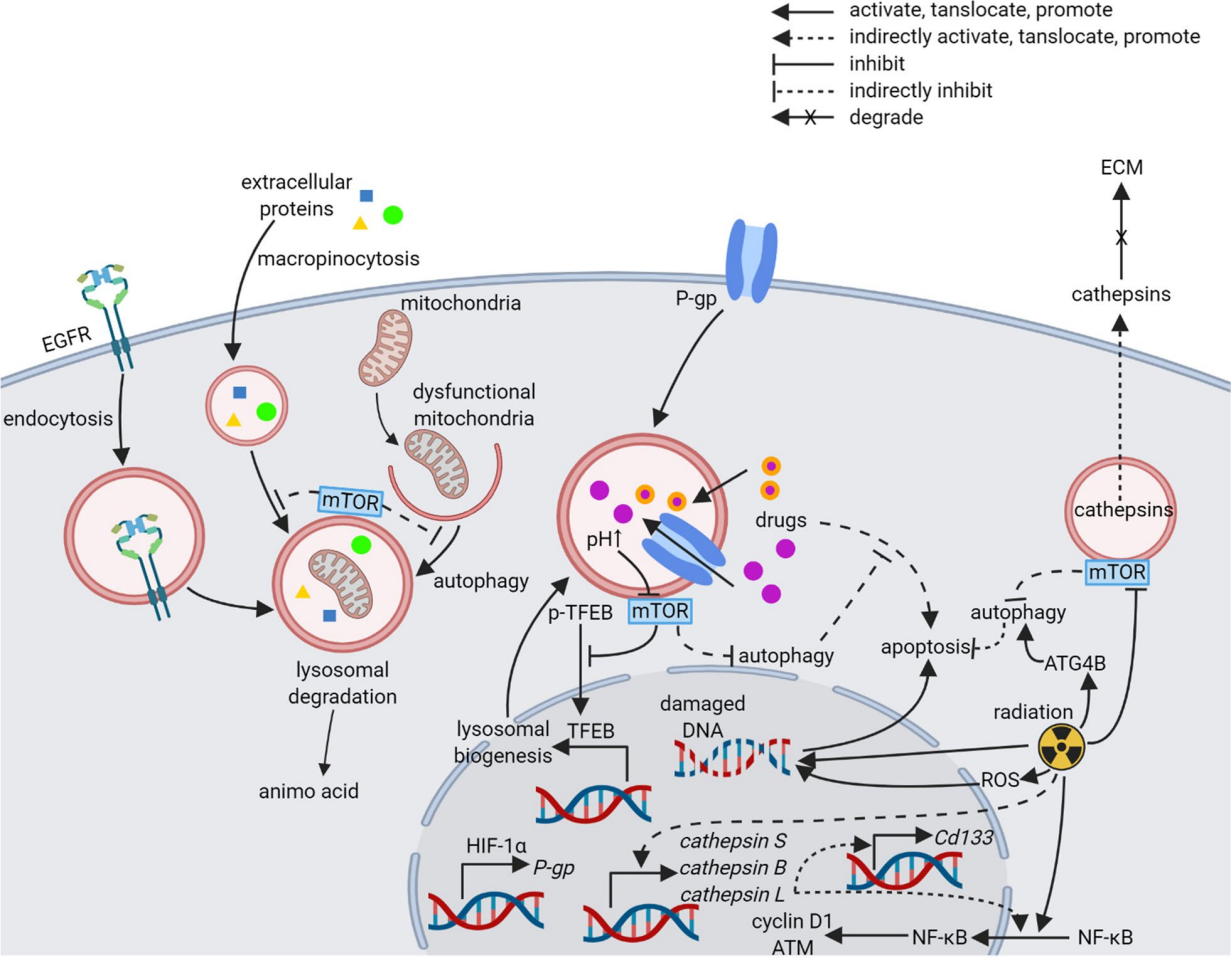
Role of lysosomes in malignant behaviors of tumor cells. Created with BioRender.com

The lysosome-related mechanisms have been summarized in Fig. 2. Interventions targeting these mechanisms seem to be promising. Interestingly, different mechanisms are found in tumor associated macrophages and other components of tumors.

### Lysosomes in tumor-associated macrophages (TAMs)

Massive infiltrated TAMs in tissues always indicate chemotherapy resistance, tumor angiogenesis, immune suppression, metastasis, and poor prognosis [238–240]. Therefore, TAMs have been a hotspot for a few decades. Polarization of TAMs has been proposed to give an outline of diverse phenotype changes of TAMs, which are able to assume distinct and metastable phenotypes in response to different environmental stimuli [241, 242]. Although more complicated spectrum of macrophage polarization have been proposed [243, 244], the paradigm of M1/M2 is now wildly used. M1 macrophages, namely activated macrophages, show pro-inflammatory phenotype, which is stimulated by ligands of TLRs, interferon gamma (IFN-γ), tumor necrosis factor alpha (TNF-α), and pathogenic microorganisms [241, 245–247]. On the contrary, transforming growth factor beta (TGF-β), interleukin-4 (IL-4), interleukin-10 (IL-10), and IL-13 induce M2 macrophages, also known as alternatively activated, macrophages that serve as anti-inflammatory factors [241, 242, 248–251]. TAMs are broadly M2-like macrophages even though they may temporarily express M1-like phenotypes at the early stage of tumor growth [252]. Lysosomes in TAMs have been shown to participate in polarization regulation, as well as additional behaviors distinct from macrophage polarization, such as immune mediation, stromal degradation, and angiogenesis.

#### Regulation of TAM polarization by autophagy

Direct regulators of macrophage polarization are transcription factors such as NF-κB, signal transducer and activator of transcription 3 (STAT3), signal transducer and activator of transcription 6 (STAT6), and hypoxia-inducible factor-2α (HIF-2α) [253–256]. Through activation or degradation of these transcription factors or their up-stream mediators, many factors take part in regulation of macrophage polarization. Lysosomal membrane proteins and luminal proteins participate in macrophage polarization by mediating autophagy, as well as other degradation activities. Alternatively, these lysosomal proteins, together with luminal ions, are able to regulate polarization through aforementioned transcription factors independently from degradation activities.

The relationship between autophagy and macrophage polarization is controversial. Many researches manifested that autophagy promoted M2-like polarization of macrophages, but several others showed opposite discoveries. In a model of colorectal carcinoma, autophagosome degradation and downstream M2-like polarization of TAMs were demonstrated to be positively related, which was mediated by cathepsin S [257]. In vitro treatment of chloroquine or knockout of cathepsin S retarded autophagic flux, increased expression of M1-type gene, inducible nitric oxide synthase (iNOS), and abrogated upregulation of M2-type genes, arginase-1 (Arg-1), found in inflammatory zone 1 (FIZZ1), and IL-10 [257]. Accordingly, in vivo experiment found that cathepsin S knockout significantly decreased tumor burden and liver metastasis [257]. A possible link between autophagy and M1-like polarization has been proposed to be degradation of NF-κB [256, 258]. Selective autophagy was involved in M2-like polarization of bone marrow-derived macrophages (BMDMs) treated with hepatoma tumor cell conditional medium [256, 258]. The conditional medium from hepatoma cell line ML-1_4a_ elevated M2-like expression pattern in BMDMs by stimulating sequestosome 1 (p62/SQSTM1) depended ubiquitination of NF-κB p65 followed by autophagy-mediated degradation of NF-κB p65 [256]. Afterwards, autophagosomes loaded with NF-κB p65 formed autolysosomes with lysosomes under the guidance of extracellular signal-regulated kinase 1/2 (ERK1/2), downstream of TLR2 signal [256]. However, M. Shan et al. reported that M2-like markers were heightened in RAW 264.7 incubated with conditional medium from mouse breast cancer cell line 4T1, and at the same time autophagy indicated by puncta of GFP-LC3 and western blot of IC3-II was inhibited [253]. The M2-like polarization could be attributed to elevated ROS from mitochondrial, which was able to be diminished by rapamycin, an autophagy inducer [253]. In vivo, stimulation of autophagy by rapamycin dampened tumor growth and metastasis in tumor-bearing mice injected with isoprenaline, an inducer of intracellular ROS [253]. The more detailed mechanism was that rapamycin-induced autophagy decreased levels of p-mTOR, ROS/p-ERK1/2 causing inhibition of downstream signal molecules pTyr705-STAT3 and HIF-1α, which attenuated isoprenaline-mediated M2-like polarization [253]. The conflicting role of autophagy in macrophage polarization could be explained by diverse substrates of autophagy in different conditions. Therefore, it should be carefully evaluated to decide whether stimulating or suppressing autophagy is beneficial in different situations.

Except for canonical autophagy, other degradation activities can also mediate TAM polarization. LC3-associated phagocytosis (LAP), a non-canonical autophagy, was positively associated with M2-like markers in TAMs [259]. In mice deficient in LC3-associated phagocytosis, stimulator of interferon genes (STING)-dependent type I IFN response in TAMs might be the key for reducing expression of CD206 in TAMs and enhancing anti-tumor effects of T cells [259]. LAMP2A-mediated selective lysosomal degradation in TAMs was another newly discovered process that regulated phenotypes of TAMs [260]. Although LAMP2A has been reported to contribute to chaperone-mediated autophagy, the author did not clarify the classification of LAMP2A-mediated degradation here. M2-like macrophage activation, immunosuppressive tumor microenvironment, and tumor growth were reversed by inhibiting LAMP2A, whose targets were found to be peroxiredoxin 1 (PRDX1) and CREB-regulated transcription coactivator 1 (CRTC1) [260]. In the context of LAMP2A knockdown, heightened PRDX1 was correlated with a higher level of H_2_O_2_ and downstream inflammatory activation. At the same time, elevated CRTC1 increased phosphorylated cAMP responsive element binding protein (p-CREB) without perturbing downstream CCAAT/enhancer-binding protein β (CEBP/β) resulting in inflammatory activation of macrophages [260]. However, a contrary finding showed that blocking selective lysosomal degradation by ATPase H^+^ transporting V0 subunit d2 (ATP6V0d2) knockout elevated M2-like markers in TAMs resulting in promoted tumor growth in tumor-bearing mice [255]. The different results can be explained by distinct degradation target, which was found to be HIF-2α here [255]. Of note, downregulation of ATP6V0d2 can also be achieved in TAMs by lactate in TME through mTORC1/TFEB pathway [255]. These results indicate that lysosomal degradation targets can vary in different contexts, thus leading to different phenotypes of TAMs. More details of the degradation processes are in need for understanding the mediation of TAM polarization.

#### Activation of TAMs by TLRs

TLRs belong to a family of pattern recognition receptors that senses invading pathogens during innate immune response. Although some specific stimulators for TLRs have been found, TLRs recognize diverse microbial and host-derived ligands physiologically. Two main localizations of TLRs in cells have been found. Roughly, TLR1, 2, 4–6, and 10 localize to plasma membrane, while TLR3, 4, 7–9, and 11–13 localize to endolysosomal membranes [6]. Downstream signals of these TLRs are transduced through either myeloid differentiation factor 88 (MyD88) or TIR-domain containing adaptor molecule (TRIF) [6]. Interestingly, TLR4 primarily activates to induce MyD88 signal at the plasma membrane, and then it is endocytosed to elicit TRIF signal [261]. These two pathways compose a network causing changes in transcription level via several transcription factors including NF-κB. Here we focus on the anti-tumor effects of ligands eliciting lysosomal TLRs. A plethora of TLR ligands have been developed to imitate the situation of innate immune response, which have shown fascinating effects in reversing M2-like phenotypes of TAMs. Both poly(I:C), a TLR3 stimulator, and LPS, classical TLR4 stimulator, activated M1 macrophages to produce antitumor nitric oxide (NO) leading to inhibited tumor growth [262]. This antitumor effect could be augmented when TLR ligands synergized with type I and II IFNs [262]. Besides NO production, LPS-treated M1-like TAMs increased lysis of ovarian carcinoma cells mediated by nature killer cells (NK cells) [263]. In addition, polyethyleneimine [264], cationic dextran [264], and multiwalled carbon nanotubes [265] also activated TLR4 signal in TAMs leading to reversed M2-like phenotypes and reduced tumor burden possibly through NF-κB. Several TLR7 ligands including CL264 [262], 1V270 [266], Gardiquimod [267], let-7b [268], and R848 [269] yielded similar impacts on M2-like TAMs. Improved anti-tumor effects of TLR7 ligands were achieved when the ligands were combined with IFNs [262], anti-programmed cell death protein 1 (PD1) antibody [266], and TGF-β receptor inhibitor [267]. Differently, TLR9 agonist, CpG ODN, potentiated tumor antigen presentation activity of TAMs, instead of M1-like polarization, causing slowed tumor growth in vivo when combined with engineered T cell transfer [270]. It is worth noting that, ligands of TLRs should be selected carefully or delivered specifically, because they may also activate TLRs in tumor cells leading to facilitated proliferation, survival, drug resistance, immunotolerance, and angiogenesis [271, 272]. At present, some TLR agonists have entered clinical trials listed in Table 4, indicating their potential capability in improving combination therapy and immunotherapy for cancer.

**Table 4:** Interventions targeting lysosomes

Disease	Intervention	Stage of development	Comment	NCT number
*Atherosclerosis*	Temsirolimus ± Dexamethasone	Phase II	mTOR inhibitor	NCT03942601
	Temsirolimus	Phase III	mTOR inhibitor	NCT04433572
	Chloroquine	Not applicable	Autophagy/lysosome inhibitor	NCT00455403
	Hydroxychloroquine	Phase IV	Autophagy/lysosome inhibitor	NCT04161339
*Alzheimer's disease*	Trehalose	Phase I	Autophagy inducer	NCT04663854
	Hydralazine	Phase III	Autophagy inducer	NCT04842552
	Rapamune	Phase I	Autophagy inducer	NCT04200911
	Rapamycin	Phase II	Autophagy inducer	NCT04629495
*Parkinson’s disease*	Exablate BBBD with Cerezyme	Not applicable	Enzyme replacement therapy	NCT04370665
*Systemic lupus erythematosus*	Hydroxychloroquine or chloroquine	Phase II	Autophagy/lysosome inhibitor	NCT01946880
	Sirolimus	Phase II	Autophagy inducer	NCT04582136
	Sirolimus	Phase II	Autophagy inducer	NCT04736953
	Rapamycin	Phase II	Autophagy inducer	NCT00779194
*Crohn’s disease*	Rapamycin	Not applicable	Autophagy inducer	NCT02675153
	Ciprofloxacin + Doxycycline + Hydroxychloroquine + Budesonide	Phase II	Combination of hydroxychloroquine with others	NCT01783106
*Rheumatoid arthritis*	Temsirolimus	Phase II	mTOR inhibitor	NCT00076206
	Sirolimus	Phase I/II	Autophagy inducer	NCT00392951
	Infliximab + DMARDs (methotrexate; chloroquine; leflunomidum; cyclosporin A; sulfasalazine; OM 89)	Phase III	Combination of chloroquine with others	NCT00521924
*Multiple sclerosis*	Temsirolimus	Phase II	mTOR inhibitor	NCT00228397
	Sirolimus	Phase I/II	Autophagy inducer	NCT00095329
*Fabry disease*	Agalsidase alfa	Phase III	Enzyme replacement therapy	NCT01298141
	Agalsidase beta	Phase IV	Enzyme replacement therapy	NCT00081497
*Tay-Sachs disease*	AXO-AAV-GM2	Phase I	Gene therapy	NCT04669535
*Mucopolysaccharidosis diseases*	Aldurazyme (for type I)	Phase III	Enzyme replacement therapy	NCT00258011
	Idursulfase (for type II)	Phase II/III	Enzyme replacement therapy	NCT00630747
	SAF-301 (for type III)	Phase I/II	Gene therapy	NCT02053064
	ABO-102 (for type III)	Phase I/II	Gene therapy	NCT04088734
	Naglazyme (for type IV)	Phase IV	Enzyme replacement therapy	NCT00299000
	elosulfase alfa (for type IV)	Not applicable	Enzyme replacement therapy	NCT03204370
	AAV2/8.TBG.hARSB (for type IV)	Phase I/II	Gene therapy	NCT03173521
	UX003 (for type VII)	Phase I/II	Enzyme replacement therapy	NCT01856218
*Pompe disease*	Alglucosidase alfa	Phase IV	Enzyme replacement therapy	NCT04676373
*Gaucher disease*	Imiglucerase	Phase IV	Enzyme replacement therapy	NCT04656600
	Velaglucerase alfa	Phase IV	Enzyme replacement therapy	NCT04718779
	taliglucerase alfa	Phase IV	Enzyme replacement therapy	NCT04002830
*Non-small cell lung cancer*	Binimetinib + Hydroxychloroquine	Phase II	Combination Therapy	NCT04735068
	Paclitaxel + Carboplatin ± Bevacizumab ± Hydroxychloroquine	Phase II	Combination Therapy	NCT01649947
	Bevacizumab + Carboplatin + Paclitaxel + Hydroxychloroquine	Phase I/II	Combination Therapy	NCT00728845
	Sunitinib + Rapamycin	Phase I	Combination Therapy	NCT00555256
	Neratinib ± Temsirolimus	Phase II	Combination Therapy	NCT01827267
*Small cell lung cancer*	Chloroquine	Phase I	Autophagy/lysosome inhibitor	NCT00969306
	Etoposide + Carboplatin + Atezolizumab + BNT411 (TLR7/8 agonist)	Phase I/II	Combination Therapy	NCT04101357
*Colon cancer*	Bupivacaine liposome suspension (for pain control)	Phase IV	Bupivacaine slowly released from lysosomes	NCT02052557
	FOLFOX/bevacizumab ± Hydroxychloroquine	Phase I/II	Combination Therapy	NCT01206530
	Vorinostat + Hydroxychloroquine	Phase II	Combination Therapy	NCT02316340
	nab-rapamycin + mFOLFOX6 and Bevacizumab	Phase I	Combination Therapy	NCT03439462
	Temsirolimus + Cetuximab	Phase I	Combination Therapy	NCT00593060
	Pembrolizumab + Poly-ICLC (TLR3 agonist)	Phase I/II	Combination Therapy	NCT02834052
	FOLFIRI + Cetuximab + IMO-2055 (TLR9 agonist)	Phase I	Combination Therapy	NCT00719199
*Breast cancer*	Hydrochloroquine	Phase II	Autophagy/lysosome inhibitor	NCT01292408
	Ixabepilone + Hydroxychloroquine	Phase I/II	Combination Therapy	NCT00765765
	Letrozole + Palbociclib + Hydroxychloroquine	Phase I/II	Combination Therapy	NCT03774472
	Chloroquine	Phase II	Autophagy/lysosome inhibitor	NCT02333890
	Zoledronic acid + Odanacatib (cathepsin K inhibitor)	Phase I/II	Combination Therapy	NCT00399802
	Trastuzumab + Rapamycin	Phase II	Combination Therapy	NCT00411788
	Inetetamab + Rapamycin + Chemotherapy	Phase III	Combination Therapy	NCT04736589
	Rapamycin	Phase II	mTOR inhibitor	NCT02642094
	Radiation + Cyclophosphamide + Imiquimod (TLR7 agonist)	Phase I/II	Combination Therapy	NCT01421017
*Hepatocellular cancer*	Sorafenib ± Hydroxychloroquine	Phase II	Combination Therapy	NCT03037437
	temsirolimus	Phase II	mTOR inhibitor	NCT01079767
	RO7119929	Phase I	TLR7 agonist	NCT04338685
*Cholangiocarcinoma*	ABC294640 ± Hydroxychloroquine	Phase II	Combination Therapy	NCT03377179
*Gastrointestinal cancer*	Cobimetinib + Atezolizumab + Hydroxychloroquine	Phase I/II	Combination Therapy	NCT04214418
*Prostate cancer*	Hydroxychloroquine	Early Phase 1	Autophagy/lysosome inhibitor	NCT02421575
	Docetaxel ± Hydroxychloroquine	Phase II	Combination Therapy	NCT00786682
	Odanacatib	Phase II	Cathepsin K inhibitor	NCT00691899
	Temsirolimus	Phase II	mTOR inhibitor	NCT00919035
	Bevacizumab + Temsirolimus	Phase I/II	Combination Therapy	NCT01083368
	Temsirolimus + Diphenhydramine	Phase II	Combination Therapy	NCT00887640
*Biliary cancer*	Trametinib + Hydroxychloroquine	Phase II	Combination Therapy	NCT04566133
*Ovarian cancer*	Temsirolimus	Phase II	mTOR inhibitor	NCT00926107
	Cisplatin + Pembrolizumab + Rintatolimod (TLR3 agonist)	Phase I/II	Combination Therapy	NCT03734692
	OC-L + Ampligen (TLR3 agonist)	Phase I/II	Combination Therapy	NCT01312389
*Pancreatic cancer*	LY3214996 ± Hydroxychloroquine sulfate	Phase II	Combination Therapy	NCT04386057
	Gemcitabine + Abraxane + Hydroxychloroquine	Phase I/II	Combination Therapy	NCT01506973
	Binimetinib + Hydroxychloroquine	Phase I	Combination Therapy	NCT04132505
	Sirolimus	Phase II	mTOR inhibitor	NCT00499486
	Bevacizumab + Temsirolimus	Phase II	Combination Therapy	NCT01010126
	Radiation Therapy + Nivolumab + SD-101 (TLR9 agonist)	Phase I	Combination Therapy	NCT04050085
	INCAGN01949 + CMP-001 (TLR9 agonist)	Phase I	Combination Therapy	NCT04387071
*Melanoma*	Dabrafenib + Trametinib ± Hydroxychloroquine	Phase II	Combination Therapy	NCT04527549
	Sorafenib + Temsirolimus	Phase I	Combination Therapy	NCT00349206
	MART-1 Antigen ± GLA-SE (TLR4 agonist)	Early Phase I	Combination Therapy	NCT02320305
	NY-ESO-1 protein + Montanide + Poly ICLC (TLR3 agonist)	Phase I/II	Vaccine	NCT01079741
*Multiple myeloma*	Bortezomib + hydroxychloroquine	Phase I	Combination Therapy	NCT00568880
*Brain neoplasms*	Temsirolimus	Phase I	mTOR inhibitor	NCT00949026
*Head and neck cancer*	Temsirolimus + Weekly Paclitaxel + Carboplatin	Phase I/II	Combination Therapy	NCT01016769
	Cetuximab + EMD 1201081 (TLR9 agonist)	Phase II	Combination Therapy	NCT01040832
*Renal cell cancer*	Sunitinib + Temsirolimus	Phase I	Combination Therapy	NCT01122615
*Bladder cancer*	Temsirolimus	Phase II	mTOR inhibitor	NCT01827943
	Sirolimus	Early Phase I	mTOR inhibitor	NCT02753309
*Glioma*	Tumor-lysate pulsed DC vaccination + adjuvant poly ICLC (TLR3 agonist)	Phase II	Vaccine	NCT01204684
*Esophageal cancer*	URLC10-177 + TTK-567 + CpG-7909 (TLR9 agonist)	Phase I/II	Vaccine	NCT00669292
*Follicular Lymphoma*	Radiation Therapy + Ibrutinib + SD-101 (TLR9 agonist)	Phase I/II	Combination Therapy	NCT02927964
*Non-Hodgkin Lymphoma*	local irradiation + CPG 7909 (TLR9 agonist)	Phase I/II	Combination Therapy	NCT00185965
*Advanced or metastatic tumor combined with COVID-19*	Avdoralimab + Monalizumab + GNS651 (autophagy inhibitor)	Phase II	Combination Therapy	NCT04333914
*Nonspecific cancer*	GSK1795091	Phase I	TLR4 agonist	NCT02798978
	Anti-Cancer Agent + SHR2150 (TLR7 agonist)	Phase I/II	Combination Therapy	NCT04588324
	Echopulse + PD-1 + Imiquimod (TLR7 agonist)	Phase I	Combination Therapy	NCT04116320
	Durvalumab + MEDI9197 (TLR7/8 agonist)	Phase I	Combination Therapy	NCT02556463
	Ipilimumab + Nivolumab + Tilsotolimod (TLR9 agonist)	Phase I	Combination Therapy	NCT04270864
	NY-ESO-1 protein + Montanide ± Resiquimod (TLR7/8 agonist)	Phase I	Vaccine	NCT00821652
	BMS 986178 + SD-101 (TLR9 agonist)	Phase I	Combination Therapy	NCT03831295

Function of several repeatedly mentioned chemical: mTOR inhibitor: Temsirolimus, Sirolimus, Rapamycin, Rapamune; autophagy/lysosome inhibitor: Hydroxychloroquine, Chloroquine. +: combination; +/−: with or without

#### Activation of TAMs by inorganic ions and pH changes

Luminal inorganic ions are another factors influencing TAM polarization. Lysosomes are characterized by low luminal pH around 4–6, which is dynamically maintained by diverse transmembrane proteins on lysosomal membrane. Thanks to the profound development of endolysosomal patch clamp technique, plenty of ion channels and transporters have been identified [273]. Basing on these findings, regulation of luminal pH and related mechanisms involving these transmembrane proteins are able to be explored. Chloroquine, a weak base agent, is able to be trapped in lysosomes, causing elevated lysosomal pH [274]. Interestingly, chloroquine switched TAMs from M2 to M1 phenotype resulting in decreased myeloid-derived suppressor cells (MDSCs) and Tregs, which enhanced anti-tumor T-cell immunity. In chloroquine treated TAMs, the elevated lysosomal pH caused Ca^2+^ release through lysosomal Ca^2+^ channel TRPML1. Released lysosomal Ca^2+^ activated p38 and NF-κB, which implemented M1 polarization of TAMs [275]. In parallel, metabolism reprogramming in TAMs from oxidative phosphorylation to glycolysis by TFEB was Ca^2+^-dependent facilitating anti-tumor effect further [275]. Likewise, hydroxychloroquine, a derivative of chloroquine, heightened M1-like molecules (iNOS, IFN-γ, IL-12b, IL-1b, and MHC II) in TAMs and induced CD8^+^ T cell infiltration and activation [276]. In accordance with these findings, an intriguing comparison of acidity between M1 and M2 polarized macrophages found phagosomes in M1 macrophages to preserve more neutral pH, greater oscillatory alkalinization, and slower acidification than phagosomes in M2 macrophages [277]. Mechanistically, the property of phagosomes in M1 macrophages was attributed to proton consumption upon superoxide generation by the nicotinamide adenine dinucleotide phosphate (NADPH) oxidase, intermittent opening of voltage-gated proton channel (H_V_1) channels, and reduced proton-pumping activity [277]. It seems that luminal pH could dynamically interact with phenotype of macrophages. Not only are interventions of luminal pH able to change phenotypes of macrophages, but also polarized macrophages demonstrate different luminal pH. Limited information about the relationship between ion channels and phenotype of TAMs are available at present. Since totally 22 ion channels have been discovered on lysosome-related vesicles, compelling findings may exist in this field. Novel inhibitors or activators of the lysosomal ion channels may provide extra access to regulating phenotype of TAMs.

#### Role of cathepsins in function of TAMs

Although polarization of TAMs is the greatest focus, other behaviors of TAMs are also of importance. As mentioned above, expression alteration of cathepsins is detected wildly in TME. The origins of cathepsins in tumor tissues have been revealed to be both tumor cells [191, 192] and TAMs [191, 278–280]. For TAMs, the versatile proteins regulate extracellular matrix degradation, angiogenesis, chemoresistance, and macrophage recruitment in TME. As a family of peptidases, cathepsins degrade not only peptide in lysosomes but also extracellular stroma. Although the extracellular environment does not provide optimal pH condition for cathepsins, cathepsins could still retain proteolytic activities. For example, cathepsin B can function as an exopeptidase at acid pH, while it has endopeptidase activity at neutral pH due to the conformational change of the occluding loop in cathepsin B [281]. Including cathepsins, other proteases such as urokinase-type plasminogen activator (uPA), tissue plasminogen activator (tPA), elastases, and matrix metalloproteinases (MMPs) form a cascade of proteolytic activation to facilitate degradation of extracellular matrix [282]. In models of breast cancer, TAM-derived cathepsin B [283], X [283], and L [284] were requisite for tumor cell invasion and metastasis. Likewise, cathepsin B [280], S [280], and X [191] from TAMs contributed to tumor cell invasion in pancreatic cancer. IL-4 has been repeatedly accused of stimulating cathepsin secretion in TAMs [254, 280, 284]. Besides secreted cathepsins, cathepsins in lysosomes of TAMs could be responsible for the digestion of internalized collagen fragment from tumor microenvironment [285]. Owning, at least in part, to extracellular matrix degradation, cathepsins augment angiogenesis. It has been reported that TAM-derived cathepsin S contributed to angiogenesis in colorectal tumor [286]. More in-depth results highlighted IL-4 to be responsible for inducing TAM-supplied cathepsins B and S to promote angiogenesis in pancreatic tumor [280]. Thus, cathepsins from not only tumor cells but also TAMs are able to promote invasion and angiogenesis.

Importantly, catalytic activity may be or may not be pivotal for the functions of cathepsins, which would elicit diverse mechanisms by different motifs [191]. In the apoptosis pathway induced by chemotherapy agents, cathepsins released from disturbed lysosomes into cytoplasm are crucial for eliciting tumor cell death [64], which is a process independent of catalytic activity [287]. However, paclitaxel increased macrophage-derived cathepsins B and S that protected tumor cells from apoptosis [279]. The effect that TAMs curtailed mammary tumor cell death induced by paclitaxel, etoposide, or doxorubicin was reversed by cathepsin inhibition [279]. I. Larionova et al. proposed a possible mechanism in their review to explain how cathepsin B from TAMs protected tumor cells. These cathepsin B elicited NF-κB in tumor cells and enhanced chemoresistance by inducing IL-1β and TNFα secretion [288]. It’s fairly reasonable, since a similar mechanism has been deciphered in MDSCs [289]. The paradoxical results on apoptosis regulation imply a variable function of cathepsins depending on their origin and location. Thus, knockdown or inhibiting TAM-derived cathepsins may be able to mitigate chemoresistance.

For macrophage recruitment, macrophage-supplied cathepsin K upregulated cyclooxygenase-2 (COX2) and chemokine ligand 2 (CCL2), two cytokines recruiting macrophages, and knockout of cathepsin K decreased macrophage infiltration [290]. Later research unraveled role of cathepsin S in the regulation of CCL2 [291]. This regulation relied on CD74, a substrate of cathepsin S, and NF-κB [291]. There seems to be a positive feedback that cathepsins from TAMs or tumor cells regulated cytokines that participated in the recruitment of more macrophages.

**Fig. 3:**
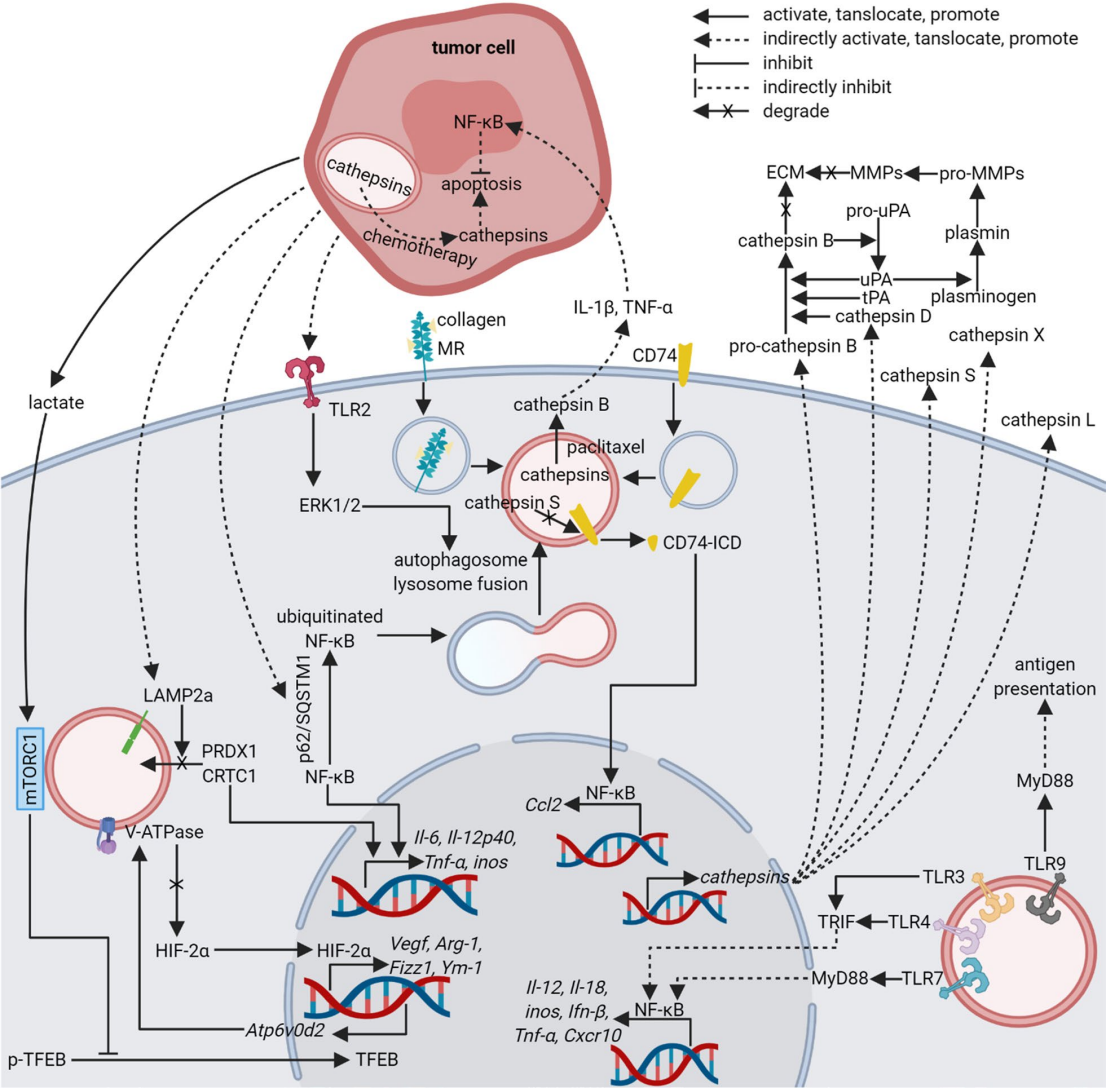
Role of lysosomes in TAMs. Created with BioRender.com

In summary, malignant behaviors of tumors are attributed, on a large scale, to M2-like polarization and several other activities of TAMs. Lysosomes are able to regulate phenotypes of TAMs with or without degradation-related mechanisms. These mechanisms are achieved based on components of lysosomes, including membrane proteins, luminal proteins and ions, which have been exhibited in Fig. 3. Potential therapy targets can be found based on the detailed understanding of these compelling mechanisms.

### Lysosomes in dendritic cells (DCs)

Besides macrophages, DCs are the other subset of antigen-presentation cells that prime CD8^+^ T cells by MHC-I or CD4^+^ T cells by MHC-II. Functions of DCs are negatively regulated by a number of factors in TME, such as IL-6, IL-10, and TGF-β [292]. Mechanistically, IL-6 reduced human leukocyte antigen (HLA)-DR surface expression levels by lysosomal protease, cyclooxygenase 2, arginase, and STAT3 activation, thus attenuating T cell stimulation [293]. Despite the mechanisms that inhibit activities of DCs, a group of LAMP3^+^ DCs in hepatocellular carcinoma have been found to possess the ability to regulate multiple T cell subsets and migrate from tumors to hepatic lymph nodes [294]. An evaluation of esophageal squamous cell carcinoma samples from 80 patients also found that infiltrating LAMP3^+^ DCs were positively correlated with intratumoral CD8^+^ T cells and favorable prognosis indicating a potential important role of LAMP3 in antigen presentation by DCs [295]. Although lysosomal proteases are involved in inhibition of HLA-DR and antigen presentation [293], conditional knockout of vacuolar protein sorting 34 (Vps34), an autophagy-related protein, in DCs caused defect in corpse-associated antigen cross-presentation to MHC-I-restricted T cells and higher incidence of lung metastases by melanoma [296]. However, classical MHC-I and MHC-II antigen presentation pathways were enhanced in Vps34 knockout DCs [296], indicating multi-facetted mediation of antigen presentation by autophagy. In summary, the role of lysosomes in DCs during infection has been extensively studied, but it remains largely fuzzy how lysosome-related mechanisms in DCs are involved in anti-tumor immunity and immune evasion in TME.

### Lysosomes in cancer-associated fibroblasts (CAFs)

In tumor, another prevalent subset of stromal cells is CAFs that interact with tumor cells through oxidative stress and NF-κB activation to secrete plenty of cytokines (e.g., IL-6, IL-8, IL-10, and IFNγ), growth factors (e.g., basic fibroblast growth factor (FGFβ) and TGFβ2), and other factors (e.g., MMP-2, MMP-9, and fibronectin) [297, 298]. Several cytokines, such as IFNγ, IL-6, IL-8, IL-10, TGFβ, and TNFα, induce autophagy in CAFs [298]. Autophagy in CAFs degrades caveolin-1, which in turn enhances autophagy in CAFs through a feed-forward mechanism [299]. Autophagy in CAFs promotes tumor progression by providing recycled nutrients, protecting from apoptosis, and inducing genomic instability in cancer cells [299, 300]. Additionally, down-regulation of breast-cancer susceptibility gene 1 (BRCA1) [301] and activation of peroxisome proliferator-activated receptor γ (PPARγ) [302] in CAFs also increases autophagy and promotes tumor growth in vivo.

Downstream mechanisms that are mediated by CAF autophagy during tumor progression include promoting chemotherapy resistance, EMT, and cancer stem cells. Contradictory associations have been reported in CAF autophagy and chemotherapy resistance of pancreatic cancer, where inhibition of CAF autophagy augmented [303] or diminished [304] anti-proliferation effect of gemcitabine. Triple-negative breast cancer cells showed potentiated migration, invasion, proliferation, and EMT after treatment with conditional medium from CAFs with a high level of autophagy, while conditional medium from CAFs pretreated with 3-Methyladenine, an autophagy inhibitor, did not have this effect [305]. Stemness of luminal breast cancer cells was enhanced depending on TLR4 stimulated by high-mobility group box 1 (HMGB1) from autophagic CAFs indicating high LC3II/TLR4 to be poor prognosis markers for breast cancer [306].

In summary, cross-talk between CAFs and tumor cells through oxidative stress, cytokines, and nutrients depend largely on autophagy resulting in more malignant behaviors of tumor cells. Disturbing autophagy in CAFs seems to be promising to control tumor progression.

### Lysosomes in T cells

In TME, T cells are the group of cells responsible for killing tumor cells. Upon recognition of tumor antigen by T-cell receptor, cytotoxic T lymphocytes release perforin and granzyme B from secretory lysosomes to kill target cells [307]. Autophagy has been revealed to influence T cell homeostasis, differentiation, function, and aging [308]. Yet, there are only a few researches focusing on the detailed role of lysosomal activities, such as autophagy, in T cell behaviors in TME.

Elevated extracellular potassium owing to necrotic cells reduced nutrient uptake in CD8^+^ T cells thereby inducing autophagy [309]. Elevated autophagy triggered metabolic and epigenetic reprogramming resulting in suppressed CD8^+^ T cell effector yet preserved stem-like behaviors including self-renewal, expansion, and multipotency, which suppressed B16 tumor growth and improved survival of mice in general [309]. Differently, another research shows that knockout of autophagy-related genes in T cells enhanced their immunity against breast cancer characterized as promotion of effector memory phenotype and IFN-γ and TNF-α production [310]. *Atg5*^*−/−*^ CD8^+^ T cells presented promoted glucose metabolism, altered histone methylation, and upregulated transcription of metabolic and effector genes [310]. The contradictory results may be explained by different baselines of control groups, in other words, high extracellular potassium in melanoma may not exist in the model of breast cancer.

As for CD4^+^ T cells, autophagy selectively degrades PU.1, the main T_H_9 cell transcription factor, and inhibited anti-tumor immunity [311]. On the contrary, genetic or pharmacological inhibition of autophagy restored anti-tumor effects by enhancing IL-9 production from T_H_9 cells [312]. Other CD4^+^ subsets were not modulated by autophagy in TME [312].

Treg cells are an immune-suppressive subset of T cells in TME. Conditional knockout of *Atg5* or *Atg7* in Treg cells impaired survival and stability of Treg cells, thus suppressing colon adenocarcinoma growth, which was underpinned by upregulated mTORC1, c-Myc, and glycolytic metabolism owing to autophagy deficiency [313]. Similar mTORC1-related hyper-glycolytic metabolism has been found in Treg cells deficient in lysosomal TRAF3-interacting protein 3 (TRAF3IP3) [314]. Knockout of TRAF3IP3 in Treg cells also booted antitumor responses [314].

In general, genetic or pharmacological inhibition of autophagy in T cells is able to promote anti-tumor immunity. The link between autophagy and alteration of metabolic states in different subsets of T cells from TME needs more intensive illustrations.

## Emerging therapy strategies targeting lysosomes

With the knowledge of various lysosome-related mechanisms in development of diseases, massive researches targeting lysosomes for therapy are reasonable. Several kinds of strategies have been proposed. First, enzyme replacement therapies are exploited for lysosomal storage disorders like GD. Second, huge amount of chemicals are able to specifically target lysosomal proteins or lysosome-related signal proteins in order to modulate behaviors of cells. Third, plenty of selective vehicles have been developed for transfecting exogenous nucleic acid or antigens to mediate expression of lysosomal proteins or elicit adaptive immunity. Finally, some nanomaterials are designed to aggregate in lysosomes for eliminating target cells. Many of these therapies have entered clinical trials, which are summarized in Table 4. Potential therapeutic interventions in pre-clinical results are listed in Table 5.

**Table 5:** Potential pre-clinical lysosome-targeted interventions for tumors

Classification	Intervention	Outcome	Reference
*Autophagy activator*	Rapamycin	Rapamycin suppresses M2 macrophage polarization	[253]
	Sorafenib	Sorafenib suppresses classical macrophage activation	[324]
*Autophagy inhibitor*	Hydroxychloroquine	Chloroquine and hydroxychloroquine switches TAMs from M2 to M1 phenotype	[275, 276]
	Chloroquine		
	Bafilomycin A1	Bafilomycin A1-induced M1-like polarization by preventing NF-κB degradation	[256]
	3-Methyladenine	3-Methyladenine prevents cross-talk between cancer cells and CAF through autophagy	[305]
*Cathepsin B inhibitor*	CA074Me	CA074Me inhibits cell invasion and inflammasome activation	[192, 289]
	CA-074	CA-074 inhibits EMT	[195]
*Cathepsin S inhibitor*	Fsn0503	Fsn0503 inhibits cell invasion	[286]
*Cathepsin L inhibitor*	Z-FY-CHO	Z-FY-CHO inhibits radio-resistance	[214]
	KGP94	KGP94 and KGP207 inhibits cancer cell invasion and M2-like TAMs	[284]
	KGP207		
*General cysteine cathepsin inhibitor*	E64	E64 inhibits cell invasion	[283]
	JPM-OEt	JPM-OEt inhibits drug resistance, tumorigenesis, angiogenesis, proliferation, and invasion	[197, 254, 279]
*Transfection vehicle*	Ghost	Ghost is applied for transfection of LAMP2A siRNA in TAMs	[260]
	PEG = MT/PC NPs	PEG = MT/PC NPs is applied for transfection of VEGF and PIGF siRNA	[330]
	Porous silicon micro-particles	Porous silicon micro-particles is applied for delivering HER2 antigen into DCs	[334]
	Lipid-coated calcium phosphate nanoparticles	Lipid-coated calcium phosphate nanoparticles is applied for delivering antigen TRP2 mRNA and PD-1 siRNA	[335]
	Metal–organic framework	Metal–organic framework is applied for delivering tumor-associated antigens into macrophages	[336]
*TAM remover*	M-chlorin	M-chlorin can enter lysosomes and delete M2 macrophages	[430]
	DOX-SPCL	DOX-SPCL concentrates in lysosomes and delete macrophages	[332]
	MEN 4901/T-0128	MEN 4901/T-0128 releases cytotoxic T-2513 in lysosomes	[333]
*TLR3 agonist*	Poly(I:C)	Poly(I:C) activates M1 macrophages	[262]
*TLR4 agonist*	LPS	LPS activates M1 macrophages	[262]
	Polyethyleneimine	Polyethyleneimine reverses M2-like polarization	[264]
	Cationic dextran	Cationic dextran reverses M2-like polarization	[264]
	Multiwalled carbon nanotubes	Multiwalled carbon nanotubes reverses M2-like polarization	[265]
*TLR7 agonist*	CL264	CL264 reverses M2-like polarization	[262]
	1V270	1V270 reverses M2-like polarization	[266]
	Gardiquimod	Gardiquimod increases M1-like polarization	[267]
	Let-7b	Let-7b reverses M2-like polarization and suppresses IL-10 production	[268]
	R848	R848 increases M1-like polarization	[269]
	R837	R837 enhances anti-tumor immunity	[338]
*TLR9 agonist*	CpG ODN	CpG ODN potentiates antigen presentation in macrophages	[270]
*Other*	Spermine modified pullulan	Spermine modified pullulan can enter lysosomes and reprogram M2 macrophages to M1	[337]

### Enzyme replacement therapies

Although lysosomal enzymes are usually synthesized in ER and modified in Golgi network, some extracellular enzymes can be taken up and delivered to lysosomes, which makes enzyme replacement therapy possible [315]. Decades ago, patients with GD were treated with glucocerebrosidase extracted from placentae. Afterward, recombinant enzymes produced by CHO cells were invented [316]. At present, enzyme replacement therapy for type 1 Gaucher disease has been a successful commercial therapy [315]. Additional clinical trials applying enzyme replacement therapies for other LSDs have been summarized in Table 4. More effective enzyme replacement therapies are expectable. However, there is still a big challenge for the application of enzyme replacement therapy, because we lack efficient methods for systematical delivery, especially across blood–brain barrier. Thus, both recombinant enzymes and delivery strategies are of great importance for enzyme replacement therapies.

### Regulating autophagy

Autophagy is the key process in the development of atherosclerosis, neurodegeneration diseases, autoimmune disorders, and tumor, especially in orchestrating macrophage phenotypes. As mentioned above, activating autophagy by Latrepirdine and CCI-779 are potential therapy methods for PD [114] and HTT [124], respectively. This complex cellular process has many feasible targets for intervention. Among the set of proteins constituting the pathway that regulates autophagy, mTOR is the central one for targeting. Table 4 shows plenty of clinical trials where mTOR inhibitors, such as temsirolimus, sirolimus, and rapamycin, are applied alone or in combination with other therapies for atherosclerosis, autoimmune disorders, and malignant tumors. Although it is well known that inhibition of mTOR would elicit autophagy, limited results are available to prove that antiatherogenic effects of mTOR inhibitors are achieved via autophagy induction [74]. Everolimus cleared macrophages in plaques through autophagy-dependent cell death, a downstream event of mTOR inhibition [317]. In fact, more researches demonstrate anti-inflammatory effect to be responsible for antiatherogenic outcome of mTOR inhibitors, such as rapamycin [318], everolimus [319], and sirolimus [320]. The anti-inflammatory effect may also be caused by balancing M1 and M2-like macrophages through mTOR intervention [70]. Moreover, two mTOR inhibitors, LY294002 and PP242, evoked transport of TFEB to nucleus [9], which would initiate lysosomal biogenesis leading to ameliorating lysosomal dysfunction [58]. Besides targeting mTOR, other agents can also modulate cell phenotypes via regulating autophagy. For example, trehalose and hydralazine might have therapeutic effects for AD through inducing autophagy (NCT04663854 and NCT04842552). By inhibiting autophagy, bafilomycin A1 [256], chloroquine [321], and 3-methyladenine [322, 323] promoted pro-inflammatory markers or suppressed anti-inflammatory markers in macrophages, which may, at least partially, underpin the huge amount of clinical trials for malignant tumors applying chloroquine or hydroxychloroquine alone or in combination with other therapies in Table 4. In contrast, anti-inflammation function of sorafenib [324], urolithin A [325], and spermine [326] are autophagy-dependent. It’s an interesting question why both autophagy stimulator, such as rapamycin, and inhibitor, such as hydroxychloroquine, are largely applied in trials for tumors. A possible explanation is the multifaceted role of mTOR pathway that not only inhibits autophagy but also regulates anabolism and proliferation [327]. An additional reason might be diverse autophagy-related mechanisms in different cell subsets at different stages. For instance, autophagy suppresses M2-like polarization of TAMs [253] but promotes survival and stability of Treg cells [313]. The positive and negative relationships between autophagy and cell death have been found in different cancer cells [232–237]. Thus, administration of autophagy inhibitors or activators should be carefully determined and specifically delivered.

### Inhibiting cathepsins

As mentioned above, cathepsins from both tumor cells and TAMs promoted malignant phenotype of tumors, including invasion, EMT, radiotherapy resistance, angiogenesis, and anti-inflammatory TME. In the laboratory, cathepsin B inhibitor, CA074Me, inhibited invasion [192] and 5FU-induced inflammasome activation that intensified growth of tumor cells [289]. Similarly, CA-074 dampened E-cadherin down-regulation induced by cathepsin B, implying its role in impeding cathepsin B-induced EMT [195]. Cathepsin S inhibitory antibody, Fsn0503, also inhibited invasion of colon adenocarcinoma cell line MC38 [286]. An unnamed cathepsin S selective inhibitor, compound 6, reduced pro-inflammatory CCL2 expression in a dose-dependent manner [291]. Z-FY-CHO, specific cathepsin L inhibitor, increased radiosensitivity in glioma cell line U251 [214, 216]. Cathepsin L/K inhibitors, KGP94 and KGP207, were able to reduce invasion of breast cancer cell line 4T1 and M2-like markers of TAMs [284]. A general cysteine cathepsin inhibitor, E64, significantly mitigated invasion of tumor cells [283]. JPM-OEt, a cell-permeable derivative of E64, diminished drug resistance [279], tumorigenesis, angiogenesis, proliferation, and invasion [197, 254]. Although a host of cathepsin inhibitors have been developed in pre-clinical researches, only a few, such as Odanacatib, have entered clinical trials. Unfortunately, the effectiveness of cathepsin inhibitors is not always satisfying. Side effects may be caused by the inhibited important physiological function of cathepsins and nonspecific inhibition [328]. Odanacatib, the only selective inhibitor that entered phase III clinical trials, discontinued due to stroke-related side effects [190]. Further cathepsin inhibitors may need specific delivery or local administration and combination with other therapies. For example, since cathepsins regulate radioresistance [214], a combination of radiotherapy with cathepsin inhibitors may potentiate therapeutic effect.

### Nucleic acid transfection

Overexpression of tumor suppressor genes or downregulation of tumor promoter genes is reasonable strategies for therapies. A large number of transfection agents have been developed nowadays, but in vivo transfection of primary cells, especially macrophages, seems to be more difficult than others. More than a decade ago, a nonviral DNA delivery system, bacterial ghost, was invented [329]. Gram-negative bacterial cells were perforated after induction by gene E from bacteriophage to create empty envelopes for DNA loading. The envelopes filled with DNA were termed bacterial ghost that was able to be engulfed by macrophages. In some ways, could DNA escape from these bacterial ghost entrapped in phagoendolysosomes. Although the details remain elusive, the genes in the plasmid DNA could be expressed in macrophages [329]. Recently, this system was applied for LAMP2A knockdown in TAMs from breast cancer, which curtailed TAM anti-inflammatory activation and tumor growth [260]. Besides bacterial ghost, a novel nanomaterial named PEG = MT/PC NPs was invented [330]. This nanomaterial with dual pH-responsiveness was able to deliver specific siRNA to knockdown vascular endothelial growth factor (VEGF) and placental growth factor (PIGF) in both TAMs and breast cancer cells. PEG = MT/PC NPs internalized in TAMs via mannose-mediated access escaped from endosome/lysosome and released siVEGF and siPIGF resulting in suppression of tumor growth and lung metastasis [330]. At present, there is a lack of clinical trials applying such delivery systems for gene transfection. The application of nucleic acid delivery systems targeting lysosomes needs to be more intensively explored.

### Removing TAMs

Since TAMs promote tumor development by too many mechanisms, some researches attempted to kill TAMs instead of reprogramming them. In order to selectively delete tumor cells and TAMs, a photosensitizer, mannose-conjugated chlorin (M-chlorin) was used in photodynamic therapy [331]. M-chlorin localized mainly in lysosomes and endoplasmic reticula revealing strong cytotoxicity for both cancer cells and M2 macrophages [331]. Modified classical chemotherapy agent, sialic acid-polyethylenimine-cholesterol modified liposomal doxorubicin (DOX-SPCL), was also able to selectively bind to TAMs, concentrate in lysosomes, and exhaust TAMs [332]. Tumor-baring mice administrated with DOX-SPCL manifested exhausted TAMs and inhibited tumor growth without significant side effect [332]. Alternatively, MEN 4901/T-0128 was designed as a cytotoxic prodrug that could release cytotoxic T-2513 after digestion by lysosomal cathepsin B at pH values ranging from 3 to 5 [333]. THP-1 derived macrophages or primary macrophages from peritoneum and spleen internalized MEN 4901/T-0128 into lysosomes and released T-2513 in the culture media, which generated sufficient concentration of T-2513 to kill human carcinoma A2780 cells [266, 333]. Further improvement of these strategies might be realized if they are combined with methods that inhibit macrophage recruitment.

### Nano/micro-particle antigen carriers

Several kinds of nano/micro-particles have been developed to carry tumor antigens into antigen presentation cells, such as DCs and macrophages. These novel particles excel at selective transport and enhanced immune stimulation. Porous silicon micro-particles developed by Xia et al. achieved prolonged early endosome localization, potentiated cross-presentation, and activated type I interferon responses when engulfed by DCs [334]. Cancer vaccines based on this micro-particles loaded with human epidermal growth factor receptor 2 (HER2) stimulated robust CD8^+^ T cell immunity against HER2^+^ mammary gland tumors in mice [334]. Besides protein, mRNA vaccine can also be loaded into nanoparticles. Wang et al. created lipid-coated calcium phosphate nanoparticles to co-deliver tyrosinase-related protein 2 (TRP2) mRNA and programmed cell death-ligand 1 (PD-L1) siRNA into DCs [335]. The cargoes can be released when calcium phosphate core is dissolved in endo-lysosomal compartment, which elicited strong specific T cell response and humoral immune response and downregulated PD-L1 in DCs in mouse model of B16F10 melanoma [335]. Another interesting nanoparticle, metal–organic framework, was fabricated in an attempt to achieve powerful delivery of tumor-associated antigens [336]. Loaded tumor-associated antigens could be released when the metal–organic framework is degraded in acidic lysosomes in macrophages. This novel nanoparticle exerted enhanced prominent antitumor outcome in B16-OVA melanoma model without obvious toxicity in mice [336]. In summary, these novel nano/micro-particles have achieved targeted delivery of antigens and strengthened immune stimulation, both of the two advantages are crucial for the development of tumor vaccines in the future.

### TLR agonists and other selective agents

In order to reverse M2-like polarization of TAMs, several compounds have been invented, and their effects are lysosome-dependent. In a mouse breast cancer model of 4T1, cationic polysaccharide spermine modified pullulan (PS) was internalized into lysosomes of macrophages and reprogramed M2 macrophages to M1, which increased CD4^+^ and CD8^+^ T cells and inhibited tumor progression [337]. Other prevalent compounds that influence phenotypes of TAMs are the family of TLRs agonists. As mentioned above, TLR3/4/7/9 are located in lysosomal membranes, whose agonists are able to elicit pro-inflammatory phenotype of TAMs. Targeting TLR7 by imiquimod (R837) which was delivered to TAMs via polymer micelles stimulated antitumor immune response [338]. In clinical trials listed in Table 4, these agonists are usually combined with chemotherapy, radiotherapy, and bio-therapy. Combination of TLR agonists with tumor antigens and anti-PD1 antibodies seems to be promising for immunotherapy.

In summary, a great deal of methods targeting lysosomes have been designed. In laboratory, these strategies have yielded promising therapeutic effects. Although a great many compounds have been developed, lysosome-targeting interventions in clinical trials are limited. Rapamycin, hydroxychloroquine, and chloroquine are the most commonly used interventions. Compelling results might be found in clinical trials applying novel lysosome-targeting interventions. Notably, the side effects and off-target effects of these compounds are the major concerns in their further application. Temporary and controllable, instead of permanent, inhibition or activation may facilitate limiting side effects, because they can minimize perturbation of important physiological function. Furthermore, specific groups, such as mannose, would help improving the specificity of these lysosome-targeting nanoparticles.

## Conclusions

In this review, we summarized physiological function of lysosomes, roles of lysosomes in the process of several diseases, and potential therapeutic methods targeting lysosomes. Although a great deal of lysosomal proteins and their functions have been illustrated, many mechanisms are still elusive. For example, it’s common sense that lysosomes are acid organelles, but knowledge about the dynamic balance of the ions between the acid luminal environment and cytoplasm is limited. Besides v-ATPase, knockout of the K+ channel, transmembrane protein 175 (TMEM175), elevated luminal pH in nutrient starvation [339], indicating a relationship between luminal K+ and H+. In fact, totally twenty-two ion channels in lysosome-related vesicles have been identified [273]. Deciphering the homeostasis of ions in lysosomes seems to be an intriguing work.

For atherosclerosis that is quite dependent on metabolic disorder of LDL-C and oxLDL, a fascinating question is whether a lipid sensing mechanism exist. The amino acid sensing by mTOR and the pivotal role of mTOR in atherosclerosis development are now well illustrated. It’s reasonable to hypothesis that a certain mean of lipid sensing with or without activation of mTOR exist. Further researches will provide us a better understanding of lipid in atherosclerosis development. For neurodegeneration diseases, although defect autophagy and potential therapeutic effects have been revealed, reliable delivery of interventions across blood–brain barrier is a big challenge.

In tumor therapy, methods targeting lysosomes have shown promising effects. Improved therapeutic outcomes may be realized when these methods are combined with other conventional therapies. It would be better if the combination is well designed. For example, combination of TLR ligands and immunotherapy improved therapy effects [266, 270]. Noteworthy, role of autophagy in tumor progression varies in different stage and different cells. Thus, interventions for inhibiting or stimulating autophagy should be selected carefully and delivered specifically.

In summary, present evidences have highlighted lysosomes to be crucial organelles in disease. More in-depth understanding of lysosome-related mechanisms would facilitate development of therapies targeting lysosomes.

## Data Availability

The materials supporting our conclusion of this review is included within the article.

## Abbreviations

v-ATPase: Vacuolar H+-ATPase
ATP: Adenosine triphosphate
LAMPs: Lysosome-associated membrane proteins
CMA: Chaperone-mediated autophagy
SNAREs: Soluble *N*-ethylmaleimide-sensitive factor activating protein receptors
TLRs: Toll-like receptors
PAMPs: Pathogen-associated molecular patterns
mTOR: Mammalian target of rapamycin
MiT/TFE: Microphthalmia/transcription factor E
TFEB: Transcription factor EB
TFEC: Transcription factor EC
TFE3: Transcription factor binding to IGHM enhancer 3
MITF: Melanocyte inducing transcription factor
mTORC1: Mechanistic target of rapamycin complex 1
GSK3β: Glycogen synthase kinase 3β
ERK: Extracellular signal-regulated kinase
MAP4K3: Mitogen-activated protein kinase kinase kinase kinase 3
PKB: Protein kinase B
PP2A: Protein phosphatase 2
TRPML1: Transient receptor potential mucolipin 1
CLEAR: Coordinated lysosomal expression and regulation
M6PRs: Mannose-6-phosphate receptors
LIMP-2: Lysosomal integral membrane protein 2
GD: Gaucher disease
GGAs: Golgi-localizing, Gamma-adaptin ear domain homology, ARF-binding proteins
ULK: Unc-51-like kinase
PI3K: Phosphatidylinositol 3-kinase
LC3B: Microtubule-associated protein 1A/B light chain 3B
VPS4: Vacuolar protein sorting 4
ALIX: ALG-2 interacting protein X
HSPs: Heat shock proteins
MHC-II: Major histocompatibility complex class II
MHC-I: Major histocompatibility complex class I
SLC38A9: Solute carrier family 38 member 9
CASTOR: Cellular arginine sensor for mTORC1
LDL: Low-density lipoprotein
LDL-C: Low-density lipoprotein cholesterol
oxLDL: Oxidized LDL
HDL-C: High-density lipoprotein cholesterol
SR-A: Scavenger receptor A
LOX-1: Lipoprotein receptor-1
Atg5: Autophagy-related 5
NLRP3: NOD-like receptor family, pyrin domain containing 3
IL-1β: Interleukin-1 beta
ROS: Reactive oxygen species
ALP: Autophagy-lysosome pathway
AD: Alzheimer's disease
PD: Parkinson’s disease
HD: Huntington disease
Aβ: Amyloid-beta
APP: Amyloid precursor protein
PS1: Presenilin 1
α-syn: Alpha­synuclein
ATP13A2: ATPase cation transporting 13A2
CAG: Cysteine, adenine, and guanine
mHTT: HTT mutants
polyQ: Polyglutamine
ALFY: Autophagy-linked FYVE protein
TNF-α: Tumor necrosis factor-α
Tregs: Regulatory T cells
LSDs: Lysosomal storage disorders
GCs: Gaucher cells
NF-κB: Nuclear factor-kappaB
IL-13: Interleukin-13
MCP-1: Monocyte chemotactic protein-1
TME: Tumor microenvironment
RTK: Receptor tyrosine kinases
EGFR: Epidermal growth factor receptor
Grb2: Growth factor receptor bound protein 2
EGF: Epidermal growth factor
RALT: Receptor-associated late transducer
EMT: Epithelial–mesenchymal transition
JNK: C-Jun amino terminal kinase
RNS: Reactive nitrogen
ATG4B: Autophagy-related 4B cysteine peptidase
LKB1: Liver kinase B1
ATM: Ataxia–telangiectasia-mutated kinase
DNA-PKcs: DNA-dependent protein kinase
P-gp: P-glycoprotein
HIF-1α: Hypoxia-inducible factor-1α
TAMs: Tumor-associated macrophages
IFN-γ: Interferon gamma
TGF-β: Transforming growth factor beta
IL-4: Interleukin-4
IL-10: Interleukin-10
STAT3: Signal transducer and activator of transcription 3
STAT6: Signal transducer and activator of transcription 6
HIF-2α: Hypoxia-inducible factor-2α
iNOS: Inducible nitric oxide synthase
Arg-1: Arginase-1
FIZZ1: Found in inflammatory zone 1
BMDMs: Bone marrow-derived macrophages
p62/SQSTM1: Sequestosome 1
LAP: LC3-associated phagocytosis
STING: Stimulator of interferon genes
LAMP2a: Lysosome-associated membrane protein type 2a
PRDX1: Peroxiredoxin 1
CRTC1: CREB-regulated transcription coactivator 1
p-CREB: Phosphorylated cAMP responsive element binding protein
CEBP/β: CCAAT/enhancer-binding protein β
ATP6V0d2: ATPase H+ transporting V0 subunit d2
MyD88: Myeloid differentiation factor 88
TRIF: TIR-domain containing adaptor molecule
NO: Nitric oxide
NK cells: Nature killer cells
PD1: Programmed cell death protein 1
MDSCs: Myeloid-derived suppressor cells
NADPH: Nicotinamide adenine dinucleotide phosphate
HV1: Voltage-gated proton channel
uPA: Urokinase-type plasminogen activator
tPA: Tissue plasminogen activator
MMPs: Matrix metalloproteinases
COX2: Cyclooxygenase-2
CCL2: Chemokine ligand 2
HLA: Human leukocyte antigen
DCs: Dendritic cells
CAFs: Cancer-associated fibroblasts
FGFβ: Basic fibroblast growth factor
BRCA1: Breast-cancer susceptibility gene 1
PPARγ: Peroxisome proliferator-activated receptor γ
HMGB1: High-mobility group box 1
TRAF3IP3: TRAF3-interacting protein 3
HER2: Human epidermal growth factor receptor 2
TRP2: Tyrosinase-related protein 2
PS: Spermine-modified pullulan
VEGF: Vascular endothelial growth factor
PIGF: Placental growth factor
M-chlorin: Mannose-conjugated chlorin
DOX-SPCL: Sialic acid-polyethylenimine-cholesterol modified liposomal doxorubicin
TMEM175: Transmembrane protein 175
NPC: Niemann-Pick type C
